# Liver Cancer Incidence and Area-Level Geographic Disparities in Pennsylvania—A Geo-Additive Approach

**DOI:** 10.3390/ijerph17207526

**Published:** 2020-10-16

**Authors:** Angel G. Ortiz, Daniel Wiese, Kristen A. Sorice, Minhhuyen Nguyen, Evelyn T. González, Kevin A. Henry, Shannon M. Lynch

**Affiliations:** 1 Cancer Prevention and Control, Fox Chase Cancer Center, Philadelphia, PA 19111, USA; angel.ortiz@fccc.edu (A.G.O.); Kristen.Sorice@fccc.edu (K.A.S.); Minhhuyen.Nguyen@fccc.edu (M.N.); Evelyn.Gonzalez@fccc.edu (E.T.G.); khenry1@temple.edu (K.A.H.); 2 Geography and Urban Studies, Temple University, Philadelphia, PA 19122, USA; tug30358@temple.edu

**Keywords:** geospatial, liver cancer, neighborhood, disparities

## Abstract

Many neighborhood socioeconomic index measures (nSES) that capture neighborhood deprivation exist but the impact of measure selection on liver cancer (LC) geographic disparities remains unclear. We introduce a Bayesian geoadditive modeling approach to identify clusters in Pennsylvania (PA) with higher than expected LC incidence rates, adjusted for individual-level factors (age, sex, race, diagnosis year) and compared them to models with 7 different nSES index measures to elucidate the impact of nSES and measure selection on LC geospatial variation. LC cases diagnosed from 2007–2014 were obtained from the PA Cancer Registry and linked to nSES measures from U.S. census at the Census Tract (CT) level. Relative Risks (RR) were estimated for each CT, adjusted for individual-level factors (baseline model). Each nSES measure was added to the baseline model and changes in model fit, geographic disparity and state-wide RR ranges were compared. All 7 nSES measures were strongly associated with high risk clusters. Tract-level RR ranges and geographic disparity from the baseline model were attenuated after adjustment for nSES measures. Depending on the nSES measure selected, up to 60% of the LC burden could be explained, suggesting methodologic evaluations of multiple nSES measures may be warranted in future studies to inform LC prevention efforts.

## 1. Introduction

According to the American Cancer Society, liver cancer (LC) has more than tripled since 1980 with mortality rates increasing by nearly 3% every year since 2000 [1]. More than 40,000 new cases and over 30,000 deaths attributed to LC are estimated for 2020 nationwide [1]. Like the U.S., liver cancer incidence rates are also on the rise in Pennsylvania, with an estimated 1870 new cases and 1270 deaths from liver cancer likely to occur in 2020, accounting for about 4% of all cancer-related mortalities in the coming year [2].

The degree of increase in liver cancer rates also vary by race/ethnicity. In the last two decades, Hispanics and Asians continue to have the highest incidence of liver cancer and in the Black population, liver cancer incidence rates are increasing rapidly, widening the gap between Whites and Blacks [3]. More specifically, in the most recent national surveys, compared to incidence rates for Non-Hispanic Whites (NHW-6.3 per 100,000), Non-Hispanic Blacks (NHB-10.2 per 100,000), Hispanics (13 per 100,000) and Asians (13.5 per 100,000) reported much higher rates of liver cancer. Further, risk factors related to liver cancer also differ by race/ethnic group [4]. NHWs were more likely to have liver cancer attributed to diabetes and obesity, Asians and Blacks to infection with hepatitis B (HBV) and C (HCV), respectively and Hispanics to alcohol-related disorders [5]. Recognizing the differential rates of liver cancer and related risk behaviors by race/ethnicity, studies have recommended focusing cancer prevention efforts on those who are Black, Hispanic, and/or those born 1950–1959 since this age group is at a higher risk for HCV infection. However, the persistent increases seen in liver cancer rates suggest these high risk populations are not being reached.

In addition to race/ethnic differences, other determinants of disparities, particularly social and economic (SES) determinants of health such as poverty, income, education, employment and access to quality healthcare, at the individual and neighborhood-levels can also impact cancer outcomes [6] and can potentially improve the identification of high risk populations. For instance, liver cancer incidence rates have been shown to be higher in individuals with less education, as well as those living in poverty, perhaps due to a lack of resources to access health care facilities [7]. Additionally, previous studies have shown that neighborhood factors can independently and jointly, along with individual-level SES factors, affect multiple cancer outcomes [8], including liver cancer risk and survival [3,9,10].

In one multilevel investigation of liver cancer, that included individual-level factors (age, gender, race/ethnicity) and neighborhood SES (nSES) measures, an association between lower nSES and higher liver cancer incidence was found [11]. This study defined nSES using the Yost index (a composite score based on the following variables: median household income; % below 200% of the poverty line, Liu education index (% aged ≥25 years with college, high school and less than high school), proportion with a blue collar job; % aged ≥16 years in the workforce without a job, median rent; median house value [11]). One of the limitations of this study is that geographic disparities were also not assessed. Identification of geographic disparities, that is, where on a map the burden of liver cancer is higher than expected, is important because it can result in narrowing down neighborhoods (and subsequently high risk populations) to target for intervention [9], thus helping to prioritize often limited resources for cancer prevention. To address this limitation, we conducted an assessment of geographic disparities in liver cancer incidence using spatial scan statistics in a prior study [9]. This study found that the incorporation of nSES indices and single nSES variables related to neighborhood stability and race/ethnicity, coupled with individual-level race/ethnicity, could lead to the identification of fewer neighborhoods to target for intervention than previous recommendations to target Black, Hispanics and those born 1950–1959 [9]. However, this study defined nSES in terms of the Townsend index (summary score of the following z-transformed variables: % with no access to a car, % of crowded households, % of rented households, % unemployed [12]). This measure did not account for other domains of nSES (i.e., education, income, etc.), which also might explain disparities.

One of the limitations of this study and neighborhood and cancer studies in general, is that the variables selected to represent nSES are often inconsistent across cancer studies. Some studies may use single variables, such as poverty or % on public assistance to represent nSES. Other studies, including the 2 studies that looked specifically at liver cancer [9,11], may develop indices or composite scores that summarize domains of education, employment, poverty, transportation and so forth of a particular neighborhood (often at the Census Tract geography) [13] using different variables to represent these domains [13]. While these indices, which are examples of nSES measures, are often assumed to have a high degree of correlation, this lack of consistency in measure selection makes inferences regarding the association of nSES with liver cancer challenging [14]. Further, the characterization of a neighborhood as high or low SES may change geospatially depending on the measure selected. For instance, a neighborhood might be considered impoverished using the poverty variable but could be categorized as middle class SES using the Yost SES index [15]. Thus, beyond knowing whether liver cancer incidence rates vary geographically, it is also important to understand how disparity-related measures, including nSES, are spatially distributed, especially for liver cancer where health disparities are known to play a major role in identifying populations at high risk.

In this study, we sought to address current limitations related to nSES measure selection in cancer studies by: (1) conducting a geospatial analysis to evaluate the geographic association of various nSES measures with liver cancer incidence rates in Pennsylvania (PA); (2) introducing a geoadditive methods approach to the liver cancer literature that allows for a comprehensive, geographic assessment of multiple nSES measures and their impact on liver cancer disparities. Specifically, we applied a Bayesian geospatial additive model [16] to determine whether neighborhoods at the Census Tract level identified as having higher than expected rates of liver cancer varied geospatially after adjusting for individual level measures related to disparity (i.e., race/ethnicity), and/or common nSES measures (i.e., a standard poverty variable, neighborhood race/ethnicity, neighborhood stability and the 2 previously studied nSES indices associated with liver cancer, Townsend Deprivation Score (TDS) and Yost index). We expanded on prior studies and, also examined 4 additional, highly cited nSES measures including the Index of the Concentration of the Extremes (ICE) [17], the Socioeconomic Position Index (SEP) [13], the multiethnic study of atherosclerosis (MESA) Index [18] and the Messer Index [19]. The purpose of this comprehensive investigation was to assess whether selection of nSES measure affected liver cancer geographic disparities and whether geospatial comparisons of nSES measures could help elucidate the contribution of disparities to the liver cancer burden in PA for future intervention planning.

## 2. Materials and Methods

### 2.1. Study Population

Liver cancer cases were obtained from the PA State Cancer Registry (PCR) from 2007–2014. The study population included all PA residents with a histologically confirmed, first primary, hepatocellular carcinoma diagnosis from 1 January 2007 to 31 December 2014 according to the International Classification of Diseases for Oncology, 3rd Edition (ICD-03). There were a total of 9460 liver cancer cases included in this analysis after excluding cases with unknown age (N = 4) or missing addresses (N = 51). The institutional review board at Fox Chase Cancer Center approved this study (IRB# 17-9031).

### 2.2. Individual-Level Measures

The individual-level variables for this study included age at diagnosis (analyzed continuously based on 13 age groups (<5, 5–9, 10–14, 15–19, 20–24, 25–29, 30–34, 35–44, 45–54, 55–64, 65–74, 75–84, ≥85)), the years of diagnosis (2007–2014), sex (male or female) and race (White, Black or African American, American Indian/Alaska Native, Asian/Pacific Islander (API) and Other).

### 2.3. Neighborhood Socioeconomic (nSES) Measures

Liver cancer cases were geocoded by study staff from the residential address at the time of diagnosis up to an assigned 2010 Census Tract identification. There were a total of 2841 unique Census Tracts in this dataset. Areas or neighborhoods in this study are defined in terms of Census Tracts, which are smaller geographic areas than counties. Census Tracts contain an average of 4000 residents, making them more ideal aggregation levels for intervention targets [20]. Area-based disparity measures at the Census Tract level were assigned to the cases and were derived from the U.S. Census and the American Community Survey (ACS) (either 5-year average data 2006–2010 for cases diagnosed between 2007 and 2010 or the 2011–2015 ACS for cases diagnosed between 2011 and 2014).

The Census Tract area-level measures of disparity include single census variables as well as nSES indices that we collectively refer to throughout the remainder of the paper as nSES measures. These nSES measures were included because they have been previously investigated in other cancer studies [21]. They include 3 neighborhood variables from the U.S. Census found to be associated with liver cancer in PA in our prior analysis: (a) (race/ethnicity (% Black); (b) migration/stability (% still living in same house as one year ago); (c) racial concentration measured using the Index of Concentration at the Extremes-Hispanic (ICE Hispanic) index [9]. In addition, we also assessed the following 7 nSES measures: (1) a standard poverty variable (% population 18 and older living below the federal poverty level (Census Tract-Poverty)) as a control measure, given this is the most commonly-used nSES variable in prior literature [22]; (2) The index of concentration at the extremes (ICE index) is a continuous measure of neighborhood affluence and poverty used in prior cancer studies [15,23] that ranges from −1 (concentrated poverty) to 1 (concentrated affluence). We calculated ICE measures using Massey’s (2001) formula [17] and instructions for the integration of neighborhood income and race/ethnicity data (Krieger et al. (2016) [24]) to calculate the index of concentration at the extremes (ICE) that compares the most privileged race/ethnic group (White) to Blacks or Hispanics across income levels [24]; (3) The Townsend Index (TDS) is a neighborhood-level economic index often used in the United Kingdom that is a composite score of the following variables: % crowding, % unemployment, % no car ownership and % renter. This measure was included given previous studies show significant associations between TDS and cancer risk, indicating higher risk among those areas with lower SES [9]; (4) The Yost index is well-cited index in cancer studies [11,25,26,27,28,29] and is comprised of the following variables: median household income; % below 200% of the poverty line, Liu education index (% aged ≥25 years with college, high school and less than high school), proportion with a blue collar job; % aged ≥16 years in the workforce without a job, median rent; median house value [11,15]; (5) The socioeconomic position index (SEP Index), is developed from a standardized z-score combining the following variables: % working class, unemployment, % below the US poverty line, low education (less than high school), expensive homes and median household income [13]; (6) The index derived from the Multiethnic Study of atherosclerosis (MESA index) is an SES measure that previously found an association between low nSES and increased cancer mortality [30]. This index is comprised of the following variables: median value of owner-occupied housing units, median household income, % households receiving interest, dividend or net rental income, % adults 25 or older with complete high school, % adults 25 or older with complete college and % employed persons 16 years of age and older in executive, managerial or professional occupations; (7) The Messer Index was created using a principal components analysis (PCA) with the following variables: % males in management and professional occupations, % crowded housing, % households below poverty level, % female-headed households with dependents, % households on public assistance and households earning <$30,000 per year estimating poverty, % no-high-school and % unemployed [19]. All independent nSES measures were analyzed as categorical variables defined by quartiles or quintiles, given nSES index measures are generally analyzed categorically in literature [9,11,13,15,17,19,23,24,25,26,27,28,29,30,31,32,33,34,35,36,37,38,39,40,41,42,43,44,45,46,47,48,49,50,51,52,53,54,55,56,57,58,59,60,61]. Associations with neighborhood variables were also added to our model as continuous variables and results were similar.

### 2.4. Statistical Analysis

The correlation between each of the nSES measures was assessed using Pearson correlation [62]. The liver case counts by Census Tract were modeled as a Poisson random variable adjusted for age, gender, year of diagnosis, race, and area-level nSES measures (Model 1 below). The Poisson model was fitted by full Bayesian inference using Markov chain Monte Carlo simulation methods that allow for random samples to be drawn from posterior distributions [63,64,65]. Using a geoadditive approach that adds and evaluates the addition of each nSES measure, we generated models based on the following equation:

(1) $$Oi\sim Poisson\left(Ei\;\lambda_{i}\right)$$

(2) $$\log\left(\lambda_{i}\right)=\eta_{i}=b_{0}+offset\left(\log\left(population\right)\right)+f_{1}\left(x_{i1}\right)\ldots +f_{k}\left(x_{ik}\right)+f_{spat}^{1}\left(s_{i}\right)+U\left(s_{i}\right).$$

A Poisson distribution is used to allow for the variability due to rare events, where Oi and Ei are the observed and expected numbers of cases respectively in area i. $\lambda_{i}$ refers to the unknown relative risk (RR) in the area that is being estimated. The RR is a ratio of observed to expected number of cases. The $\eta_{i}$ references the number of liver cancer cases for a Census Tract (i) aggregated by diagnosis year, gender, age group and race. The model intercept, $b_{0}$ quantifies the average relative risk across all Census Tracts in the state. The offset was the natural logarithm of the tract-level population in each age, gender, year, and race. In addition to the relative risk, the model also includes the terms $f_{1}\left(x_{r1}\right)\ldots f_{k}\left(x_{rk}\right)$ to present a function for covariate k (e.g., each stratum specific individual and nSES measure in the model), a random effect or unstructured heterogeneity $\left(U\left(s_{i}\right)\right)$ and $f_{spat}^{1}\left(s_{i}\right)$ the structured spatial component representing the spatial dependence between areas. The unstructured effect was defined using a random effect ($U\left(s_{i}\right))$ [65]. The structured spatial component is defined as a stationary Gaussian random field specification based on the adjacency matrix defined using neighboring Census Tracts (weights based on rook’s case) [16,66]. The spatial function $f_{spat}^{1}\left(s_{i}\right)$ allows us to estimate the geographic variation for the risk of liver cancer, defined in terms of a relative risk (RR) ratio, that is based on whether spatially smoothed relative risk (using bivariate penalized spline) in a Census Tract are higher or lower compared to the statewide average of expected risk, after controlling for various covariates (e.g., individual and nSES measures ($f_{k}\left(x_{rk}\right)$)). In the model, cluster-specific random effects can be added to the usual predictors of regression models both to account for correlations between observations within clusters (i.e., tracts) and to account for unstructured heterogeneity. In the case of high spatial heterogeneity or small numbers, no smoothing is applied [67]. More broadly, Markov chain Monte Carlo (MCMC) simulation techniques, corresponding to full Bayesian inference which includes specifying prior distributions for all unknown parameters, were used to estimate RR from the Poisson regression model specified above. For this analysis, positive hyperparameters (a = b = 0.001), which assumes an inverse gamma distribution, were chosen to ensure the propriety of the joint posterior [68].

For each model, 10,000 iterations were run with the first 2000 samples used as a burn-in. Every 10th sample from the remaining 8000 samples was saved and these samples were used to construct the posterior distribution for each of the parameter estimates in the model. The 95% credible intervals (CI) were calculated based on the posterior distribution of the 800 samples to identify significant relative risks and the Census Tracts with statistically significant higher or lower rates compared to the statewide average. All models were implemented with R (R Core Team, Vienna, Austria) [69] using packages BayesX [65], BayesXsrc [63] and R2BayesX [64]. A default chain convergence specification of 1 was applied to the models. The BayesX package monitors chains and gives error messages when models fail to converge. All models converged [65]. The modeling output from the BayesX package includes the posterior means and 95% credibility intervals for the fixed effects, unstructured random effects, structured spatial effects and spatial variance. The exponentiated spatial structured effect for each Census Tract (defined in terms of RR) was mapped, providing a geographic visualization of variation in incidence rates. Census Tracts identified as having significantly higher and significantly lower RR in comparison to the statewide average were identified and mapped using 95% credible intervals to determine significance [9].

Four main sets of models were analyzed in this study based on the equation presented above:

- Model 1: individual-level model ((diagnosis year, gender, age at diagnosis, race covariates); Supplementary Material Figure S3);
- Model 2: Model 1 + neighborhood-level measures as recommended by Lynch et al. [9] ((% Non-Hispanic Black (% NHB), Hispanic ICE, Neighborhood Instability); Supplementary Material Figure S4);
- Models 3–9: Model 2 + each additional nSES measure separately: Poverty measure (Poverty—Model 3), ICE-Income (Model 4), TDS (Model 5), Yost Index (Model 6), SEP (Model 7), MESA (Model 8) and Messer Index (Model 9) presented in Supplementary Material Figures S5–S11;
- Models 10–11: Model 2 + combinations of nSES measures (TDS + Yost (Model 10), ICE Income + Yost (Model 11)). We present these two combination of index measures given they demonstrated differences in geographic variation and had the most promising results (Supplementary Material Figures S12 and S13). We also tested additional combination models, which are presented in Supplementary Material Figures S14 and S15).

Next, model comparisons and assessments were performed using Deviance Information Criterion (DIC), Geographic Disparity (GD) [15,70] and Relative Risk Range (RR Range). The DIC is a statistical measure of model fit and is defined as the sum of the posterior expected deviance and the effective number of parameters. A lower value of DIC suggests a better model fit [71]. The GD is represented as:

(3) $$GD=\left(e^{\sqrt{\sigma_{spatial}^{2}}-1}\right)\times 100.$$

The GD is a measure of the spatial variability shown as a percentage, which can be derived from Poisson models in the BayesX Package [65,66]. The GD is a description of the unexplained (remained) spatial variance ($\sigma_{spatial}^{2})$ after adjustments for an independent factor. The spatial variance was extracted from the modeling output. Higher GD assumes larger geographic disparities in the study area and suggests that more geographic variability remained unexplained after adjusting for independent variables. The RR Range shows the interval of the risk values (smallest RR to largest RR across the entire state) and is used for model comparison where a tighter range is indicative of attenuating (and subsequently helping to explain) spatial disparities in local (Census Tract) incidence rates. All maps were created using QGIS Version 3.10 (Open Source Geospatial Foundation, Beaverton, OR, USA) [72] and analyses were conducted using R version 3.6.1 (R Core Team, Vienna, Austria) [69].

## 3. Results

The total study population included 9460 cases of LC. Table 1 shows a summary of individual- and neighborhood-level characteristics. Average patient age was about 65 years and roughly three-quarters of the study population was male. The majority of diagnosed patients were White (77%) followed by Black (18%), Asian (3.4%) and Other (1.6%).

Assessing nSES measures, over 90% of patients lived in Census Tracts with a low percentage of NHB residents, 80% lived in neighborhoods with low instability and 20% of patients resided in Census Tracts with a high concentration of NHW, while 34% lived in the areas with a high concentration of Hispanics. Among the seven nSES measures compared in the analysis, the proportion of patients living in low SES Census Tracts ranged from 24–32%. Further, correlation analyses showed a high degree of statistical correlation (>80%) for the following indices: MESA & Messer, ICE Income & Yost Index, Yost Index & MESA, ICE Index & MESA and Townsend & Messer (See Supplementary Material Figure S1 for correlation matrix). However, noticeable differences in spatial variation were observed across indices (Supplementary Material Figure S2).

### 3.1. Geographic Clustering and Spatial Effects of nSES Measures: Models 1 and 2

**Figure 1:**
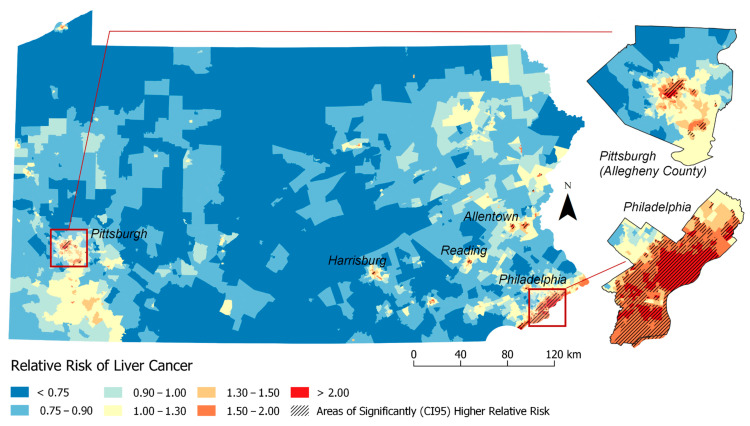
Relative Risk Estimates for Liver Cancer by Census Tract Adjusted for Individual-level factors only (Model 1: Adjusted for: individual-level factors (age + gender + year + race)). RR >1 indicates elevated risk of liver cancer incidence. Shaded areas indicate significant clusters of higher than expected rates of liver cancer based on the 95% credible interval (CI 95).

**Figure 2:**
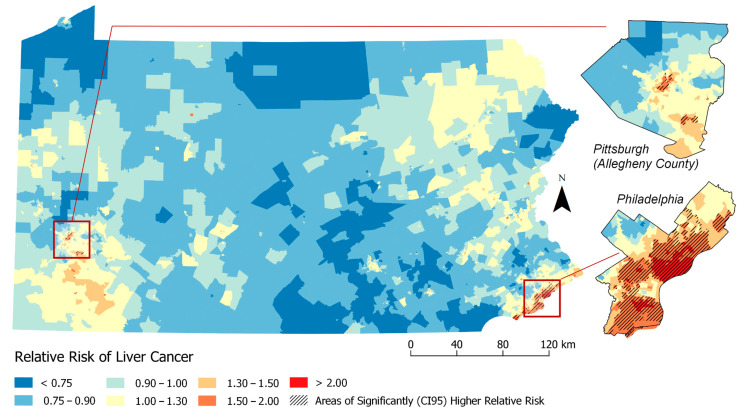
Relative Risk Estimates for Liver Cancer by Census Tract (Model 2. Adjusted for: individual-level factors + previous neighborhood variables (% Non-Hispanic Black (% NHB), Hispanic ICE and Neighborhood Instability).

The map in Figure 1 show the RR estimates for each Census Tract, as well as the Census Tracts or clusters of tracts with statistically significantly higher than expected rates of liver cancer based on our individual-level model (Model 1). Figure 2 displays Model 2 (individual plus previous neighborhood measure model). A total of 370 (12%) Census Tracts were detected in the high risk clusters in Pennsylvania in Model 1 (Figure 1) and 195 (6%) were identified in Model 2 (Figure 2).

Prior to adjusting for nSES measures, we observed clusters in Philadelphia, Allentown, Reading, Easton and Pittsburgh. The characteristics of the cases within these high risk clusters versus outside these significantly high risk clusters from Model 1 estimates are presented in Table 2. The high-risk clusters have a higher proportion of cases that are Black (43.7% in high-risk clusters vs. 10.0% outside high-risk clusters), API (6.3% vs. 2.6%) and or other races (3.3% vs. 1.2%) and a lower proportion of Whites (46.7% vs. 86.1%) compared to Census Tracts outside (i.e., lower or state average relative risk). Similarly, individual Census Tracts in high-risk clusters also had a higher proportion of Blacks, API and others races and more patients living below the poverty level (27.4% vs. 10.9%) compared to Census Tracts outside the high risk cluster. This pattern continues when comparing nSES measures of cases in high-risk clusters to those outside high-risk clusters. High-risk clusters had higher proportion of study subjects living in neighborhoods with the highest levels of Hispanic concentration (84% vs. 19%), neighborhood instability (32% vs. 17%) and deprivation (TDS (83% vs. 18%), Yost (60% vs. 14%), SEP (71% vs. 18%), MESA (69% vs. 18%) and Messer (78% vs. 19%)) compared to outside clusters. Thus, these findings suggest that nSES measures could play a strong role in identifying geographic disparities.

### 3.2. Geo-Additive Model Comparisons and Assessments

Referring to Table 3, we next compared model fit (DIC), GD and RR ranges between Models 1 and 2 with enhanced Models 3–9 that included additional nSES measures in order to determine which measures alone or in combination could help to explain observed geographic disparities in liver cancer rates (i.e., the tighter the RR range, the smaller the DIC, the smaller the GD, the better). RR ranges were more precise in Model 2 (0.48–3.44) compared to Model 1 (0.37–4.03), the DIC was lower (87,427 vs 88,005) and the %GD decreased (58.5% vs 76.5%). Models adjusted for additional nSES measures (Models 3–9) all had decreased geographic disparity and tighter RR range compared to Model 2. Comparing the seven separate nSES models, GD, RR ranges and DICs were relatively similar. The indices with the greatest reductions in geographic disparity were ICE Income (GD = 39.98%) and Yost (GD = 40.71%); the tightest RR ranges were ICE-Income (RR Range = 0.58–2.53) and Messer (RR Range = 0.56–2.48); the best model fit was SEP (DIC = 87,393), followed by Model 2 (DIC=87,427). We also explored whether combining more than one nSES measure would improve assessments (Models 10–11; See Supplementary files). Models which included combinations of nSES measures had the greatest reductions in geographic disparity using Townsend/Yost (GD = 38.53%) and ICE-Income/Yost (GD = 37.84%); the tightest RR ranges were in MESA/Messer (RR Range = 0.57–2.48) and ICE-Income/Yost Index (RR Range = 0.57–2.42); and the best model fit was in Townsend/Yost (DIC = 87,543) (Supplementary File—Table S1).

**Figure 3:**
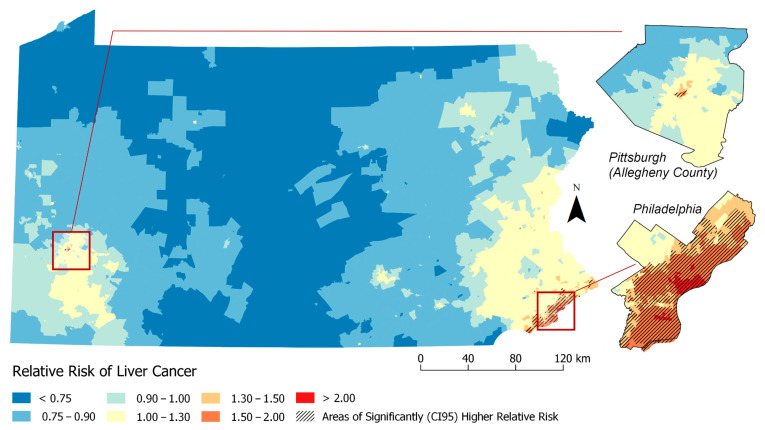
Relative Risk Estimates for Liver Cancer by Census Tract (Model 4. Adjusted for: individual-level factors + previous neighborhood variables (%Non-Hispanic Black (%NHB), Hispanic ICE and Neighborhood Instability) + ICE-Income).

**Figure 4:**
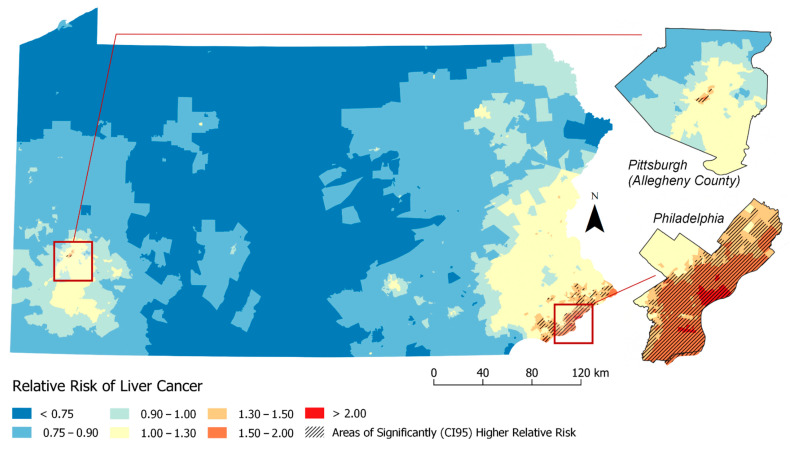
Relative Risk Estimates for Liver Cancer by Census Tract (Model 11. Adjusted for: individual-level factors + previous neighborhood variables (% Non-Hispanic Black (% NHB), Hispanic ICE and Neighborhood Instability) + ICE-Income/Yost).

Based on Table 3 results, Figure 3 and Figure 4 visualizes geographic disparities in liver cancer rates when adjusting for the ICE-income measure (Model 4) and combined ICE-Income/Yost measure (Model 11). These maps were displayed because these nSES measures were able to explain the highest percentage of liver cancer geographic disparities and they were able to show a marked attenuation in liver cancer rates. For instance, when comparing Figure 1-Model 1 to Figure 3-Model 4 (ICE-Income) to Figure 4-Model 11 (ICE-Income/Yost), a progressive attenuation (i.e., less dark red areas) with each additional nSES measure is noted, particularly in the Philadelphia and Pittsburgh areas.

## 4. Discussion

This study used geospatial Bayesian models to identify neighborhood clusters of higher than expected LC incidence rates in PA. We found several neighborhoods or clusters of Census Tracts with significantly higher rates of LC incidence than expected that were not explained by individual-level characteristics including age, gender and race. Building on a previous investigation of liver cancer disparities in PA [9], we found that the addition of any nSES measure to spatial models helped explain additional high risk clusters, more than models adjusted for individual-level characteristics alone. This was demonstrated by the reduction in the geographic disparity measure (i.e., increase in spatial variance explained) and attenuation of liver cancer rates across the State of PA. There was a high degree of correlation among evaluated nSES measures and they performed similarly in geospatial models (Table 3). However, we were able to determine upon evaluation (Figure 1, Figure 2, Figure 3 and Figure 4) that ICE measures (i.e., ICE-Income) and a combined Yost/ICE-Income model consistently explained the largest portion of geographic disparities in LC rates in PA and attenuated LC rates slightly more than the other nSES measures, including poverty, the most commonly used measure to evaluate nSES.

Our overall finding that low nSES is associated with increased risk of liver cancer (Table 2) is consistent with prior, non-spatial investigations [11] that used the Yost index. However, while previous studies have identified associations between ICE measures and breast cancer in both non-spatial [23] and spatial investigations [15], to our knowledge, this is the first study to report associations with LC. ICE with race adjustments (e.g., ICE-Hispanic) is meant to capture racial concentration [73,74]. Similar to a previous investigation of LC, we found a strong association between LC rates and geographic distribution of LC rates in PA and ICE-Hispanic measure [9]. Prior investigations have also demonstrated that LC rates and pathways to LC often vary by race/ethnicity. For instance, Hispanics have one of the highest LC rates compared to the White population and are more likely to develop LC due to alcohol disorders compared to Whites (who often develop LC due to metabolic syndromes, that is, diabetes/obesity) [4,5]. Thus, while we adjust for race at the individual level in this analysis, it is possible that ICE-Hispanic is serving a surrogate for Hispanic ethnicity. We also found associations between the ICE income measure and LC risk. ICE-Income accounts for economic variation at extremes within a neighborhood [23,24,73,74] and our analysis suggests that more detailed variables, like ICE-income, may be a more informative nSES measure than standard neighborhood-level poverty.

Using a comprehensive Bayesian approach that expands traditional regression methods and allows for the consideration of spatial effects, we found that geographic disparities were most explained statistically (i.e., the GD was the lowest and RR were most attenuated) when considering the Yost Index with the ICE-Income index. This combination provided only a slight 2% improvement over the ICE-income measure alone, which recorded a GD of 39.9%. At first this seemed surprising, given the Yost Index and ICE-Income measure appear to have high statistical correlation (Supplementary Material Figure S1), suggesting they represent nSES similarly. However, each measure does capture different disparity-related domains. For instance, the Yost index is a composite score comprised of variables representing poverty, education, income, employment and housing [11,15]. ICE-Income is meant to capture social polarization [15,23,24,73,74]. Additionally, when visualizing these measures on a map, each measure does designate Census Tracts as high or low SES a bit differently (Supplementary Material Figure S2), suggesting that when combined, they could provide a more comprehensive understanding of LC geographic disparities. Interestingly, when we look more specifically at persistent clusters in Philadelphia and Pittsburgh, the rates of LC go down but the number/size of the high risk clusters in this area increase, compared to the ICE-Income measure alone. This could be because adjusting for both these indices could lead to neighborhoods becoming more similar to one another after adjustments. Thus, visually assessing how neighborhood risk changes with the inclusion/exclusion of different nSES measures could provide additional insight into specific factors that affect disease development (i.e., housing may have more of an impact in one area versus another). Both approaches (statistics and geospatial mapping) appear to be informative for nSES measures selection. Thus, both assessments are likely needed to improve understanding of LC disparities, to identify populations at high risk for LC and to inform whom and where to target future cancer-related interventions.

There were several limitations in our study. Our study was conducted in PA and results might not be generalizable to other states. The neighborhood and disease rate data were based on Census Tract units but it is possible results could change if findings were analyzed at a different geographic level [73]. Additionally, scaling differences for nSES measures made comparisons across indices challenging. For example, the TDS, SEP and MESA indices were all created using a z-transform, while the Yost, SEP and Messer indices were created using a principal component analysis (PCA). Further, our analysis was limited to nSES deprivation indices and it is possible that other neighborhood disparity measures related to distance to quality health care [75], social disorder [76] and physical/built environment [77] could also play a role in LC disparities and should be evaluated in future studies.

Our study supports the role of nSES measures in liver cancer and provides additional evidence to support multilevel theories that suggest cancer rates are affected by both individual and neighborhood SES factors [8]. The geospatial method used in this analysis is robust allowing its application across various cancer sites and geographic levels internationally. Results suggest multiple nSES measures should be assessed statistically and geospatially given significant clusters continued to persist in both the Philadelphia and Pittsburgh areas, even after adjustments for nSES. This suggests that unmeasured behavioral factors related to liver cancer including Hepatitis B (HBV) and Hepatitis C viral (HCV) infections, diabetes, obesity [78,79] and alcohol consumption [80], should also be explored in future studies.

## 5. Conclusions

We introduced a Bayesian geoadditive approach to study liver cancer geographic disparities. We identified clusters in Pennsylvania with higher than expected LC incidence using these non-linear Bayesian geospatial models adjusted for individual-level factors and compared them to models with nSES measures. We found that neighborhood SES index measures help to explain liver cancer geographic disparities more than individual factors alone. Relative risk ranges and geographic disparities were attenuated after adjustment for each nSES measure, particularly for models including the index of concentration at the extremes(ICE)-income and Yost measure. Over 60% of the LC burden can be explained with these two measures and it is likely that these results are applicable other states with similar urban and rural landscapes as PA. However, given the variation in percentage of disparities explained across nSES measures, future studies should consider evaluating multiple nSES measures both statistically and geospatially in order to determine which measure or group of measures could be impacting observed disease disparities. These comprehensive assessments could then lead to data-driven cancer control efforts that would allow for improvements in resource allocation and a reduction in liver cancer disparities.

## Figures and Tables

**Table 1:** Pennsylvania Liver Cancer Patient Characteristics (N = 9460).

Characteristics		Total (N = 9460)
Individual-level	**Age at diagnosis Mean years (SD)**	65.3 (12.8)
		**N (%)**
	**Gender**	
	Male	6810 (72.0%)
	Female	2650 (28.0%)
	**Race/Ethnicity**	
	White	7310 (77.3%)
	Black or African American	1659 (17.5%)
	American Indian/Alaskan Native	11 (0.1%)
	Asian/Pacific Islander (API)	324 (3.4%)
	Other	156 (1.6%)
Neighborhood-level
	**Census Tract %NHB**	
	Low <80%	8710 (92.1%)
	High ≥80%	749 (7.9%)
	Unknown	1 (0.0%)
	**Hispanic ICE (quartiles)**	
	1 High concentration of NHW	1910 (20.2%)
	2	2053 (21.7%)
	3	2292 (24.2%)
	4 High concentration of Hispanics	3200 (33.8%)
	Unknown	5 (0.1%)
	**Neighborhood Instability (quartiles)**	
	Low Neighborhood Instability	7567 (80.0%)
	High Neighborhood Instability	1893 (20.0%)
	**Poverty (quartiles)**	
	1 High SES	2136 (22.6%)
	2	2216 (23.4%)
	3	2243 (23.7%)
	4 Low SES	2863 (30.3%)
	Unknown	2 (0.1%)
	**ICE Income (quartiles)**	
	1 Concentrated affluence	2080 (22.0%)
	2	2223 (23.5%)
	3	2329 (24.6%)
	4 Concentrated poverty	2823 (29.8%)
	Unknown	5 (0.1%)
	**Townsend Index (quartiles)**	
	1 High SES	2009 (21.2%)
	2	2132 (22.5%)
	3	2250 (23.8%)
	4 Low SES	3064 (32.4%)
	Unknown	5 (0.1%)
	**Yost Index (quintiles)**	
	1 High SES	1638 (17.3%)
	2	1808 (19.1%)
	3	1824 (19.3%)
	4	1789 (18.9%)
	5 Low SES	2293 (24.2%)
	Unknown	108 (1.1%)
	**SEP Index (quartiles)**	
	1 High SES	2056 (21.7%)
	2	2199 (23.2%)
	3	2340 (24.7%)
	4 Low SES	2843 (30.1%)
	Unknown	22 (0.2%)
	**MESA Index (quartiles)**	
	1 High SES	2792 (29.5%)
	2	2461 (26.0%)
	3	2264 (23.9%)
	4 Low SES	1942 (20.5%)
	Unknown	1 (0.01%)
	**Messer Index (quartiles)**	
	1 High SES	1969 (20.8%)
	2	2207 (23.3%)
	3	2218 (23.4%)
	4 Low SES	3065 (32.4%)
	Unknown	1 (0.01%)

Abbreviations: SD, Standard deviation; ICE, Index of Concentration at the Extremes; SES, Socio-economic status; SEP, Socio-economic position; MESA, Multi-Ethnic Study of Atherosclerosis. Neighborhood Instability: % still living in same house as one year ago.

**Table 2:** Individual and Neighborhood Level Characteristics of Liver Cancer Cases.

Characteristics	% Within Statistically Significant High Risk Clusters for LC		% Outside Statistically Significant High Risk Clusters for LC		% Statewide	
	Cases N_Cases_ = 2111	Area (Census Tract) N_CT_ = 366	Cases N_Cases_ = 7349	Area (Census Tract) N_CT_ = 2851	Cases N_Cases_ = 9460	Area (Census Tract) N_CT_ = 3217
**White**	46.7	35.3	86.1	82.8	77.3	77.4
**Black or African American**	43.7	39.1	10.0	8.13	17.5	11.6
**American Indian/Alaskan Native**	0.1	0.2	0.1	0.1	0.1	0.12
**Asian/Pacific Islander (API)**	6.3	5.51	2.6	2.45	3.4	2.8
**Other**	3.3	4.86	1.2	3.52	1.6	3.68
**% Living Below Poverty**	27.4	28.7	10.9	12.1	14.6	14.0
**nSES Measures**	**Cases only n (%)** **N = 2111**		**Cases only n (%)** **N = 7349**		**Cases only n (%)** **N = 9460**	
**%Non-Hispanic Black (%NHB)**						
Low <80%	1555 (73.7%)		7155 (97.4%)		8710 (92.1%)	
High ≥80%	555 (26.3%)		194 (2.6%)		749 (7.9%)	
**Hispanic ICE (quartiles)**						
1 Concentrated affluence of NHW	24 (1.1%)		1886 (25.7%)		1910 (20.2%)	
2	62 (2.9%)		1991 (27.1%)		2053 (21.7%)	
3	248 (11.7%)		2044 (27.8%)		2292 (24.2%)	
4 Concentrated poverty of Hispanics	1776 (84.1%)		1424 (19.4%)		3200 (33.8%)	
**Neighborhood Instability (quartiles)**						
Low Neighborhood Instability	1428 (67.6%)		6139 (83.5%)		7567 (80.0%)	
High Neighborhood Instability	683 (32.4%)		1210 (16.5%)		1893 (20.0%)	
**Poverty (quartiles)**						
1 High SES	50 (2.4%)		2086 (28.4%)		2136 (22.6%)	
2	165 (7.8%)		2051 (27.9%)		2216 (23.4%)	
3	335 (15.9%)		1908 (26.0%)		2243 (23.7%)	
4 Low SES	1560 (73.9%)		1303 (17.7%)		2863 (30.3%)	
**ICE Income (quartiles)**						
1 Concentrated affluence	49 (2.3%)		2031 (27.6%)		2080 (22.0%)	
2	208 (9.9%)		2015 (27.4%)		2223 (23.5%)	
3	372 (17.6%)		1957 (26.6%)		2329 (24.6%)	
4 Concentrated poverty	1481 (70.2%)		1342 (18.3%)		2823 (29.8%)	
**Townsend Index (quartiles)**						
1 High SES	14 (0.7%)		1995 (27.1%)		2009 (21.2%)	
2	62 (2.9%)		2070 (28.2%)		2132 (22.5%)	
3	282 (13.4%)		1968 (26.8%)		2250 (23.8%)	
4 Low SES	1752 (83.0%)		1312 (17.9%)		3064 (32.4%)	
**Yost Index (quintiles)**						
1 High SES	38 (1.8%)		1600 (21.8%)		1638 (17.3%)	
2	178 (8.4%)		1630 (22.2%)		1808 (19.1%)	
3	217 (10.3%)		1607 (21.9%)		1824 (19.3%)	
4	407 (19.3%)		1382 (18.8%)		1789 (18.9%)	
5 Low SES	1257 (59.5%)		1036 (14.1%)		2293 (24.2%)	
**SEP Index (quartiles)**						
1 High SES	113 (5.4%)		1943 (26.4%)		2056 (21.7%)	
2	168 (8.0%)		2031 (27.6%)		2199 (23.2%)	
3	314 (14.9%)		2026 (27.6%)		2340 (24.7%)	
4 Low SES	1503 (71.2%)		1340 (18.2%)		2843 (30.1%)	
**MESA Index (quartiles)**						
1 High SES	80 (3.8%)		1862 (25.3%)		1942 (20.5%)	
2	217 (10.3%)		2047 (27.9%)		2264 (23.9%)	
3	351 (16.6%)		2110 (28.7%)		2461 (26.0%)	
4 Low SES	1462 (69.3%)		1330 (18.1%)		2792 (29.5%)	
**Messer Index (quartiles)**						
1 High SES	29 (1.4%)		1940 (26.4%)		1969 (20.8%)	
2	163 (7.7%)		2044 (27.8%)		2207 (23.3%)	
3	273 (12.9%)		1945 (26.5%)		2218 (23.4%)	
4 Low SES	1645 (77.9%)		1420 (19.3%)		3065 (32.4%)	

**Table 3:** Model Characteristics Comparing Model Fit (DIC), Relative Risk Range (RR) and Unexplained Geographic Disparity (GD) Percentage.

Models	DIC	GD	RR Range
Model 1 (Individual-level)	88,005	76.45%	0.37–4.03
Model 2 (Individual-level + previous neighborhood variables)	87,427	58.49%	0.48–3.44
Model 3 (Model 2 + Poverty)	87,608	47.56%	0.55–2.86
Model 4 (Model 2 + ICE-Income)	87,606	39.98%	0.58–2.53
Model 5 (Model 2 + Townsend)	87,513	48.19%	0.52–2.69
Model 6 (Model 2 + Yost Index)	87,558	40.71%	0.55–2.55
Model 7 (Model 2 + SEP)	87,393	54.91%	0.44–3.05
Model 8 (Model 2 + MESA)	87,591	42.25%	0.56–2.68
Model 9 (Model 2 + Messer)	87,535	43.02%	0.56–2.46
Model 10 (Model 2 + Townsend + Yost Index)	87,543	38.53%	0.59–2.42
Model 11 (Model 2 + ICE-Income + Yost Index)	87,617	37.84%	0.57–2.44

Abbreviations: DIC, deviance information criterion (model fit), lower values are better fit; GD, geographic disparity: square root of the spatial variance; disparity in relation to statewide relative risk average; lower number represent reduced disparities; RR Range, relative risk range previous neighborhoods variables; ICE, Index of Concentration at the Extremes; SES, Socio-economic status; SEP, Socio-economic position; MESA, Multi-Ethnic Study of Atherosclerosis; CT, Census Tracts; individual-level: age, sex, year of diagnosis, race; previous neighborhood variables: % Non-Hispanic Black (% NHB), Hispanic ICE and Neighborhood Instability.

## Supplementary Materials

The following are available online at https://www.mdpi.com/1660-4601/17/20/7526/s1, Figure S1: Pearson Correlation and Scatter Plots for Each Neighborhood Socioeconomic Measures (nSES), Figure S2: Spatial Variation Across Neighborhoods socioeconomic measures (nSES): Comparison of Discrepancies between neighborhood Census Tract identified in the highest quartile of poverty (“high poverty”) vs the highest quartile of the each nSES measure (“high deprivation”), Figures S3–S15: Relative Risk Estimates for Liver Cancer by Census Tract Maps, TableS1: Model Characteristics Comparing Model Fit (DIC), Relative Risk Range (RR) and Unexplained Geographic Disparity (GD) Percentage.
