# Supplementary material for: Liver Cancer Incidence and Area-Level Geographic Disparities in Pennsylvania—A Geo-Additive Approach
Source: Int J Environ Res Public Health. 2020 Oct 16;17(20):7526. doi: 10.3390/ijerph17207526 (PMC7588924; doi:10.3390/ijerph17207526)
Supplement: Supplementary file 1 [file ijerph-17-07526-s001.pdf]

Supplementary Material

Figure S1: Pearson Correlation and Scatter Plots for Each Neighborhood Socioeconomic Measures (nSES)

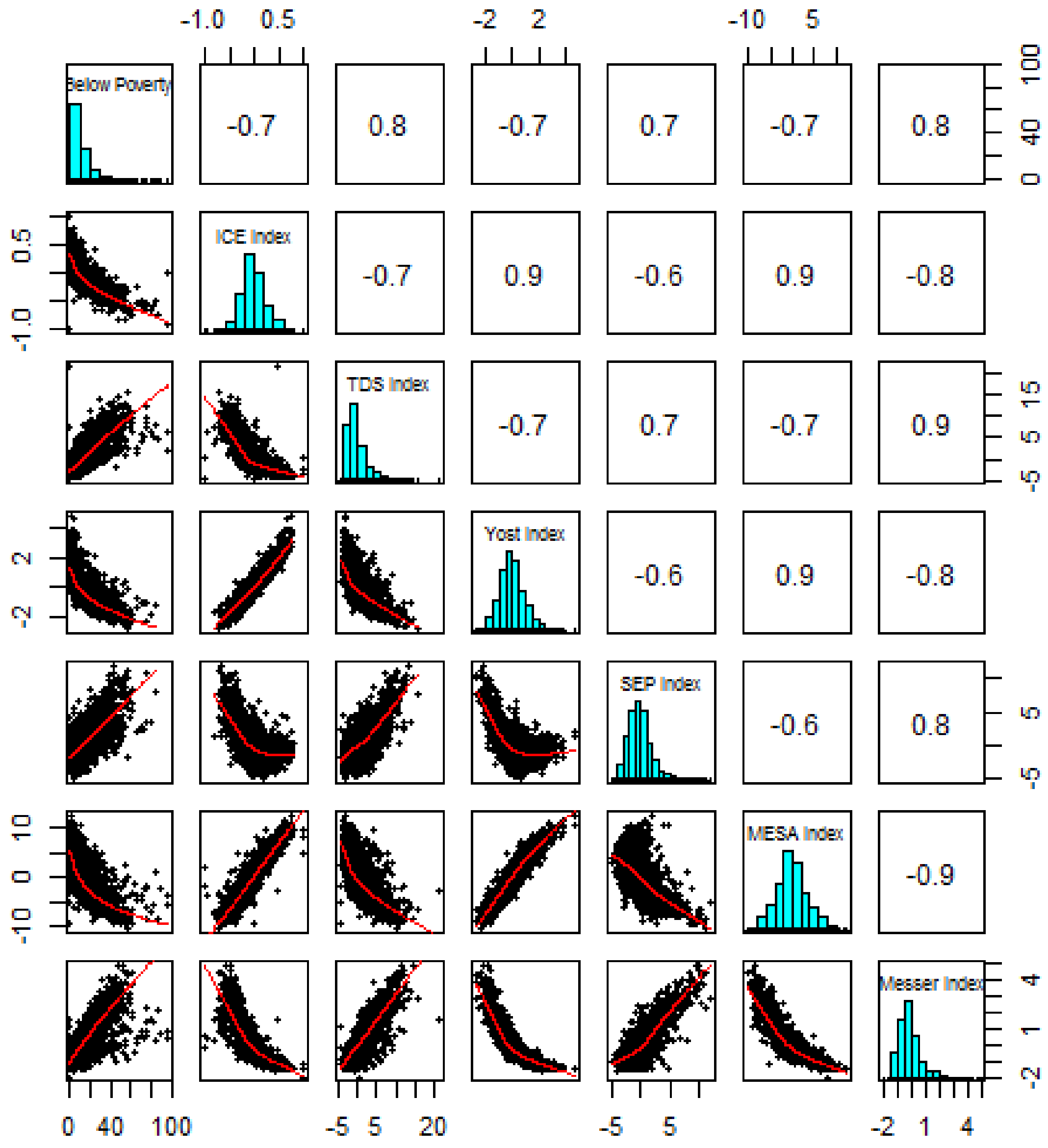

Note: Correlation values and scatterplots were used to compare the relationship between the nSES measures used in the study. The Pearson correlation was used for continuous scores with the highest correlation observed among MESA & Messer (-0.9), ICE Income & Yost (0.9), Yost & MESA (0.9), ICE Income & MESA (0.9), and Townsend & Messer (0.9). Measures which were least associated included: ICE Income & SEP (-0.6), Yost & SEP (-0.6), and SEP & MESA (-0.6).

Figure S2: Spatial Variation Across Neighborhoods socioeconomic measures (nSES): Comparison of Discrepancies between neighborhood census tract identified in the highest quartile of poverty ("high poverty") vs the highest quartile of the each nSES measure ("high deprivation")

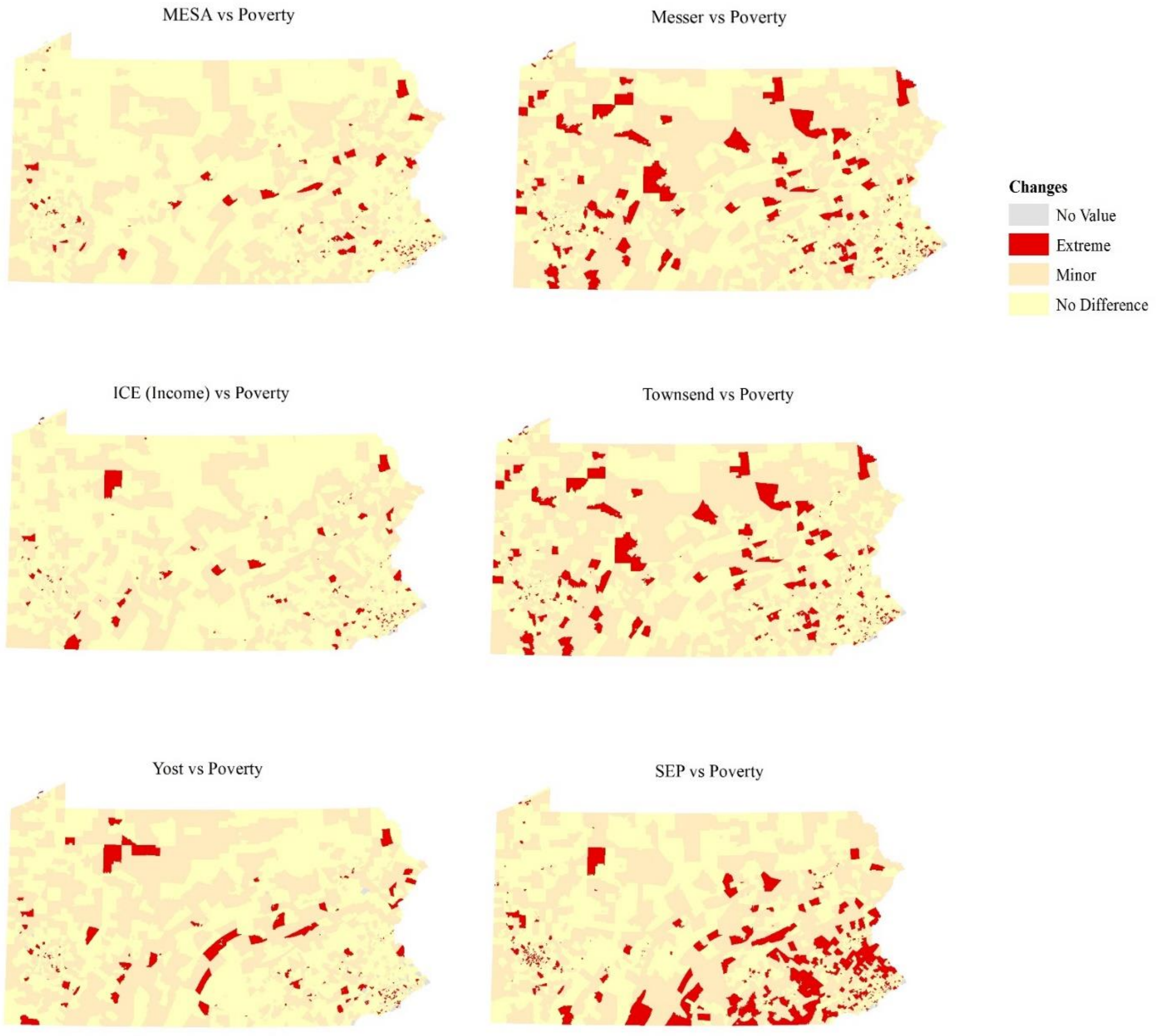

Note: Spatial comparisons of nSES measures were assessed in the maps above. All measures were standardized to quartiles for comparison purposes. The difference in quartile was used as an indicator to determine variation between the reference nSES measure, poverty, and the other 6 indices. If quartile values were equal between the two measures in a CT, then there was no difference, a 1 quartile difference was minor, and differences in 2 or greater were labelled extreme.

Figure S3. Relative Risk Estimates for Liver Cancer by Census Tract Adjusted for Individual-level factors only (Model 1: Adjusted for: individual-level factors (age + gender + year + race)). RR>1 indicates elevated risk of liver cancer incidence. Shaded areas indicate significant clusters of higher than expected rates of liver cancer based on the 95% credible interval (CI95); N = 370 Census Tracts within statistically significant high risk clusters for LC

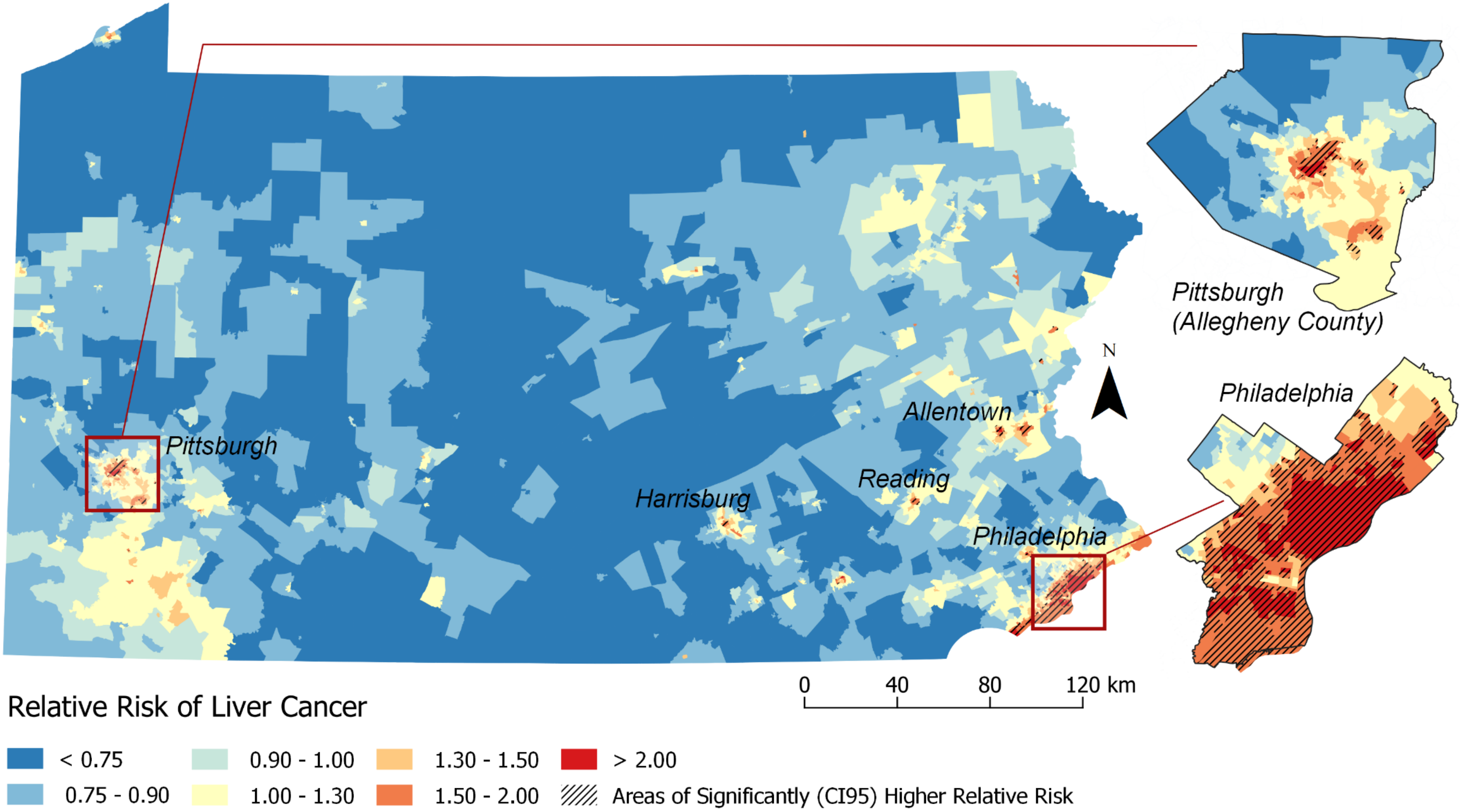

Figure S4. Relative Risk Estimates for Liver Cancer by Census Tract (Model 2. Adjusted for: individual-level factors + previous neighborhood variables (%Non-Hispanic Black (%NHB), Hispanic ICE, and Neighborhood Instability)); N = 195 Census Tracts within statistically significant high risk clusters for LC

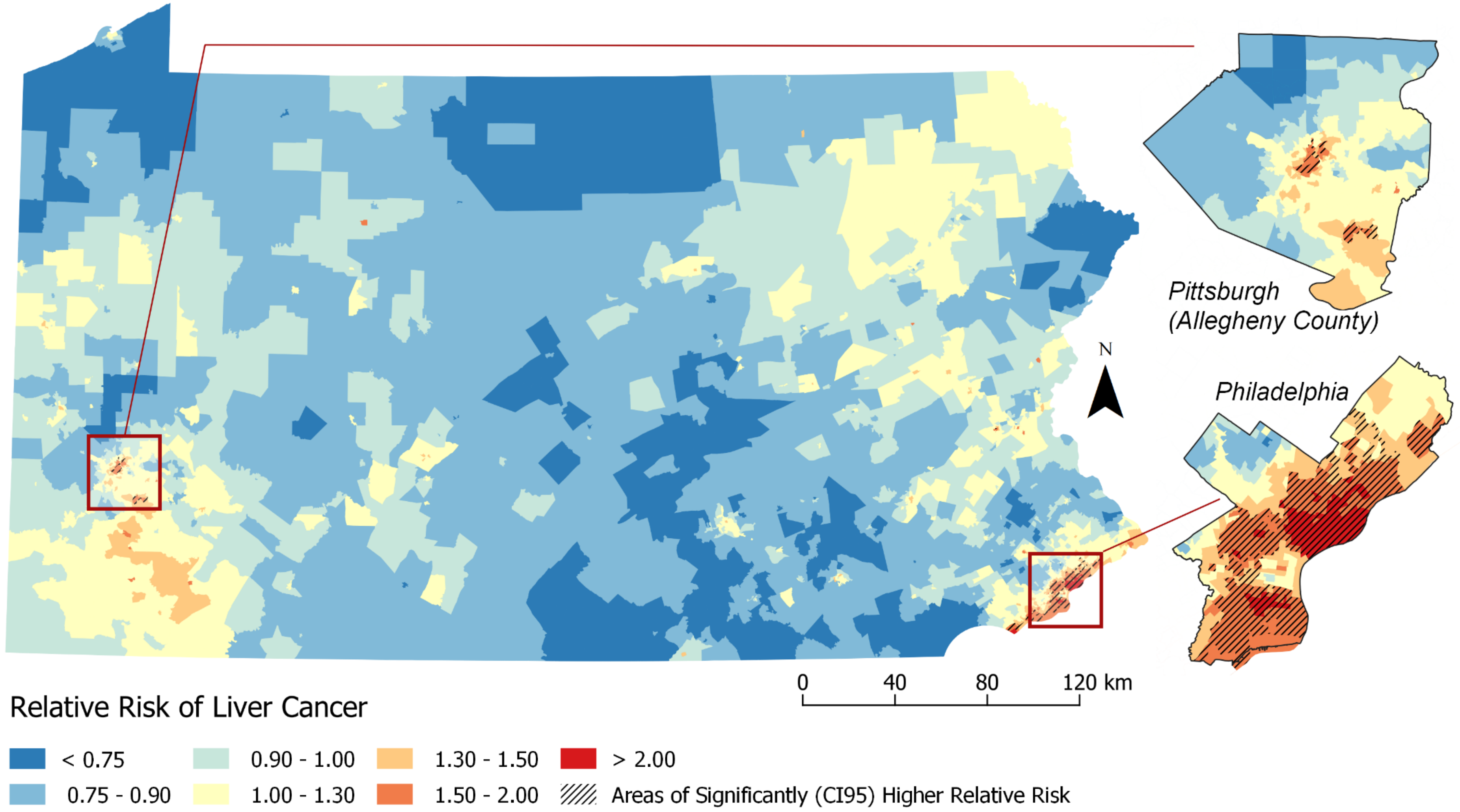

Figure S5. Relative Risk Estimates for Liver Cancer by Census Tract (Model 3. Adjusted for: individual-level factors + previous neighborhood variables (%Non-Hispanic Black (%NHB), Hispanic ICE, and Neighborhood Instability) + Poverty); N = 221 Census Tracts within statistically significant high risk clusters for LC

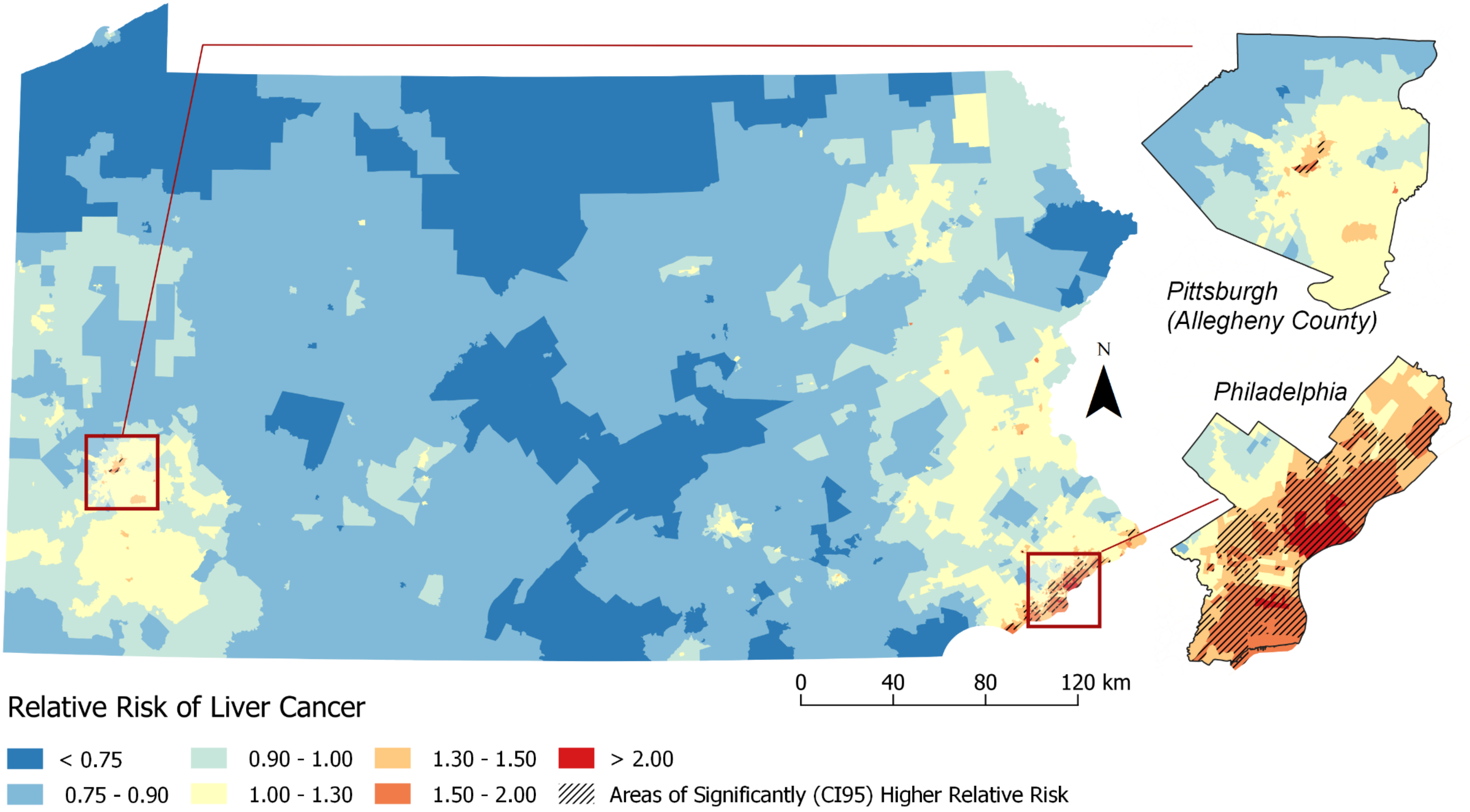

Figure S6. Relative Risk Estimates for Liver Cancer by Census Tract (Model 4. Adjusted for: individual-level factors + previous neighborhood variables (%Non-Hispanic Black (%NHB), Hispanic ICE, and Neighborhood Instability) + ICE-Income); N = 319 Census Tracts within statistically significant high risk clusters for LC

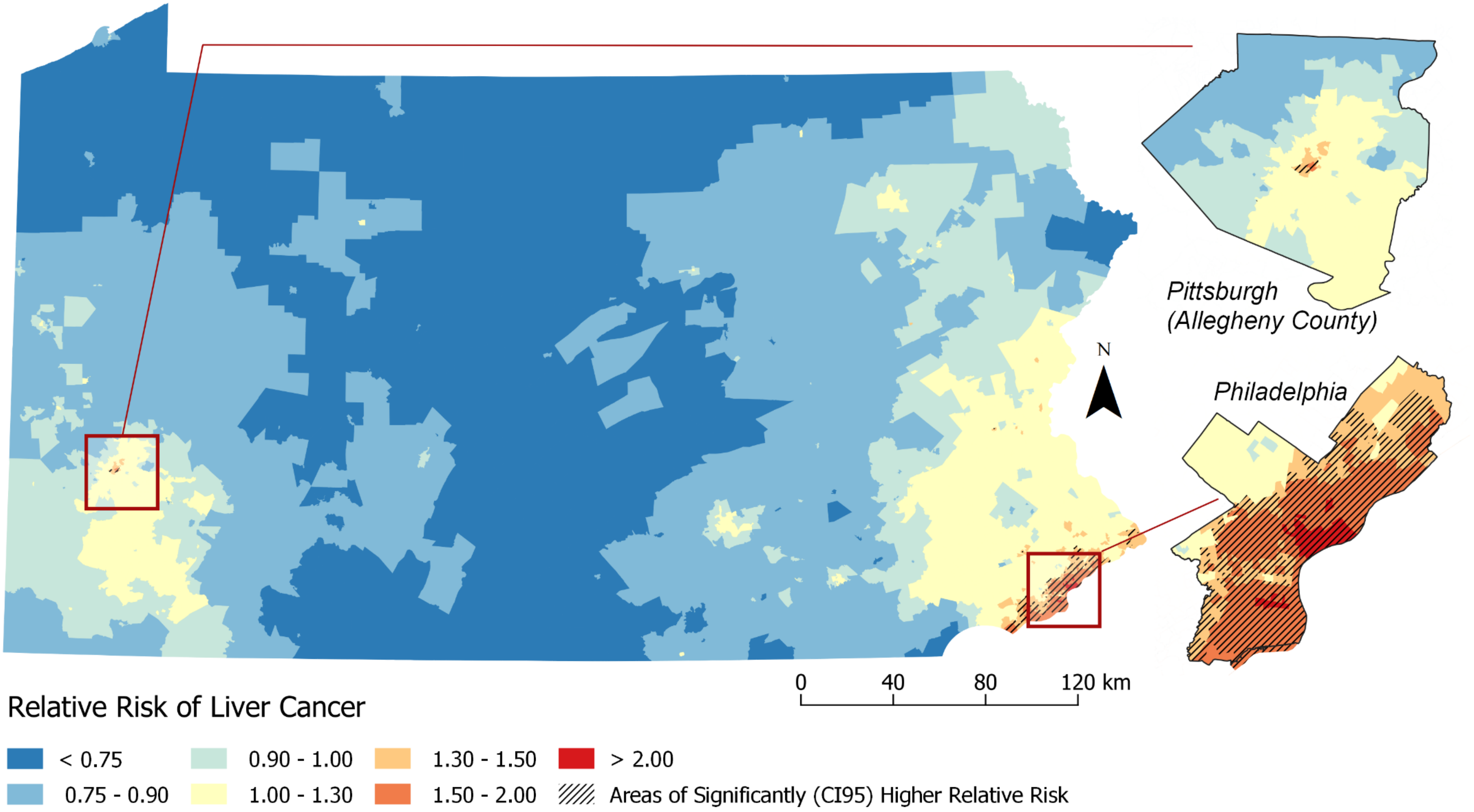

Figure S7. Relative Risk Estimates for Liver Cancer by Census Tract (Model 5. Adjusted for: individual-level factors + previous neighborhood variables (%Non-Hispanic Black (%NHB), Hispanic ICE, and Neighborhood Instability) + Townsend); N = 177 Census Tracts within statistically significant high risk clusters for LC

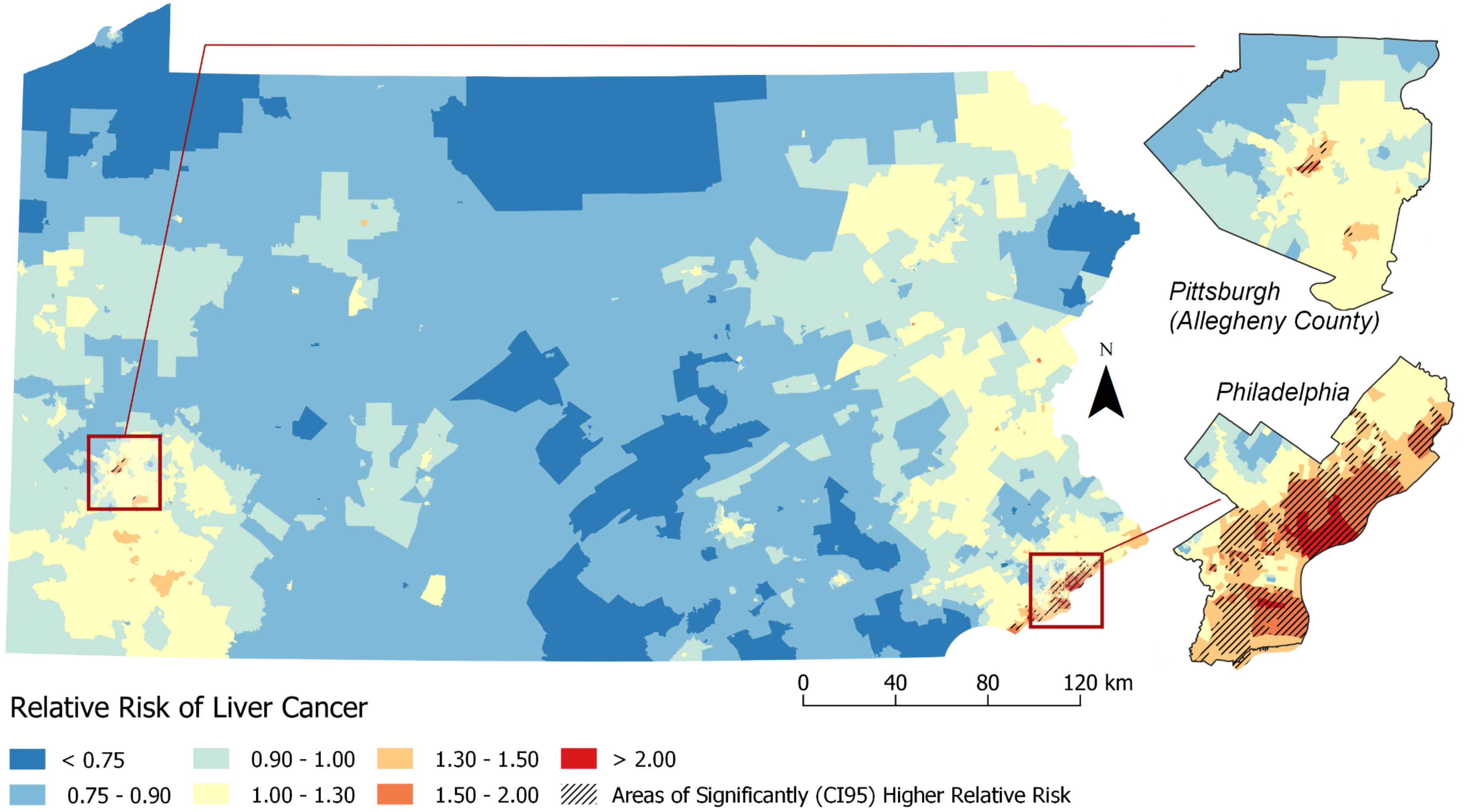

Figure S8. Relative Risk Estimates for Liver Cancer by Census Tract (Model 6. Adjusted for: individual-level factors + previous neighborhood variables (%Non-Hispanic Black (%NHB), Hispanic ICE, and Neighborhood Instability) + Yost Index); N = 342 Census Tracts within statistically significant high risk clusters for LC

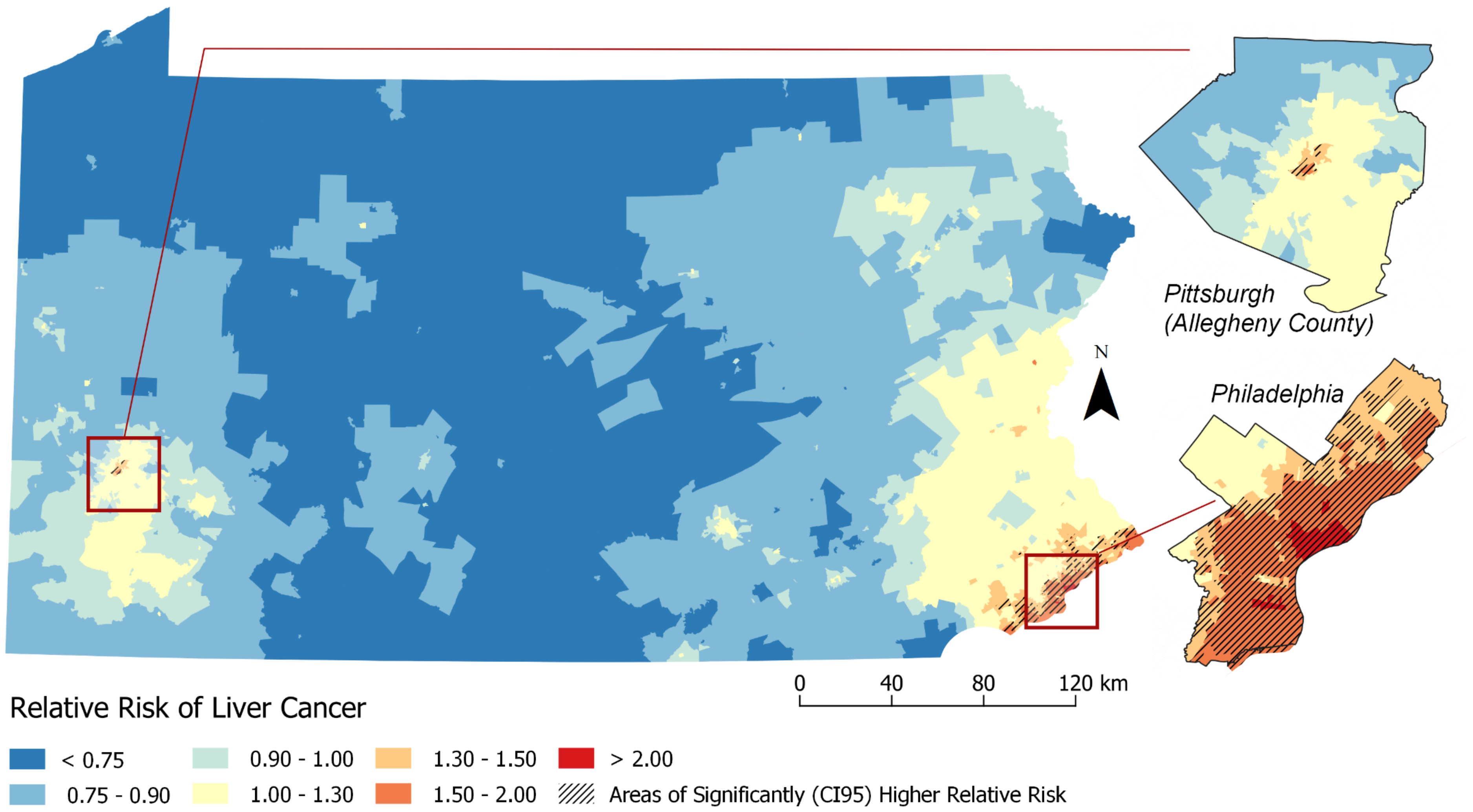

Figure S9. Relative Risk Estimates for Liver Cancer by Census Tract (Model 7. Adjusted for: individual-level factors + previous neighborhood variables (%Non-Hispanic Black (%NHB), Hispanic ICE, and Neighborhood Instability) + SEP); N = 221 Census Tracts within statistically significant high risk clusters for LC

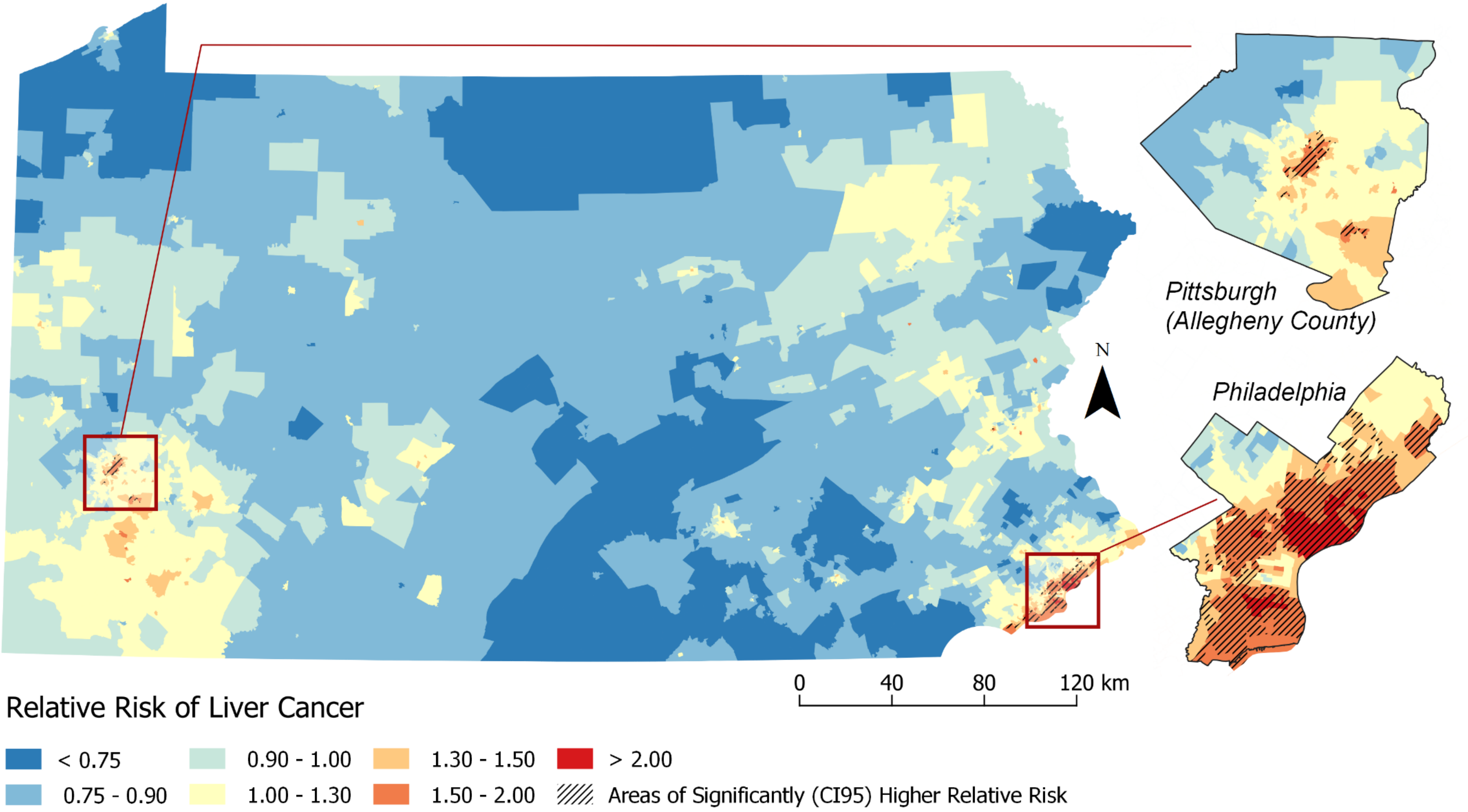

Figure S10. Relative Risk Estimates for Liver Cancer by Census Tract (Model 8. Adjusted for: individual-level factors + previous neighborhood variables (%Non-Hispanic Black (%NHB), Hispanic ICE, and Neighborhood Instability) + MESA); N = 291 Census Tracts within statistically significant high risk clusters for LC

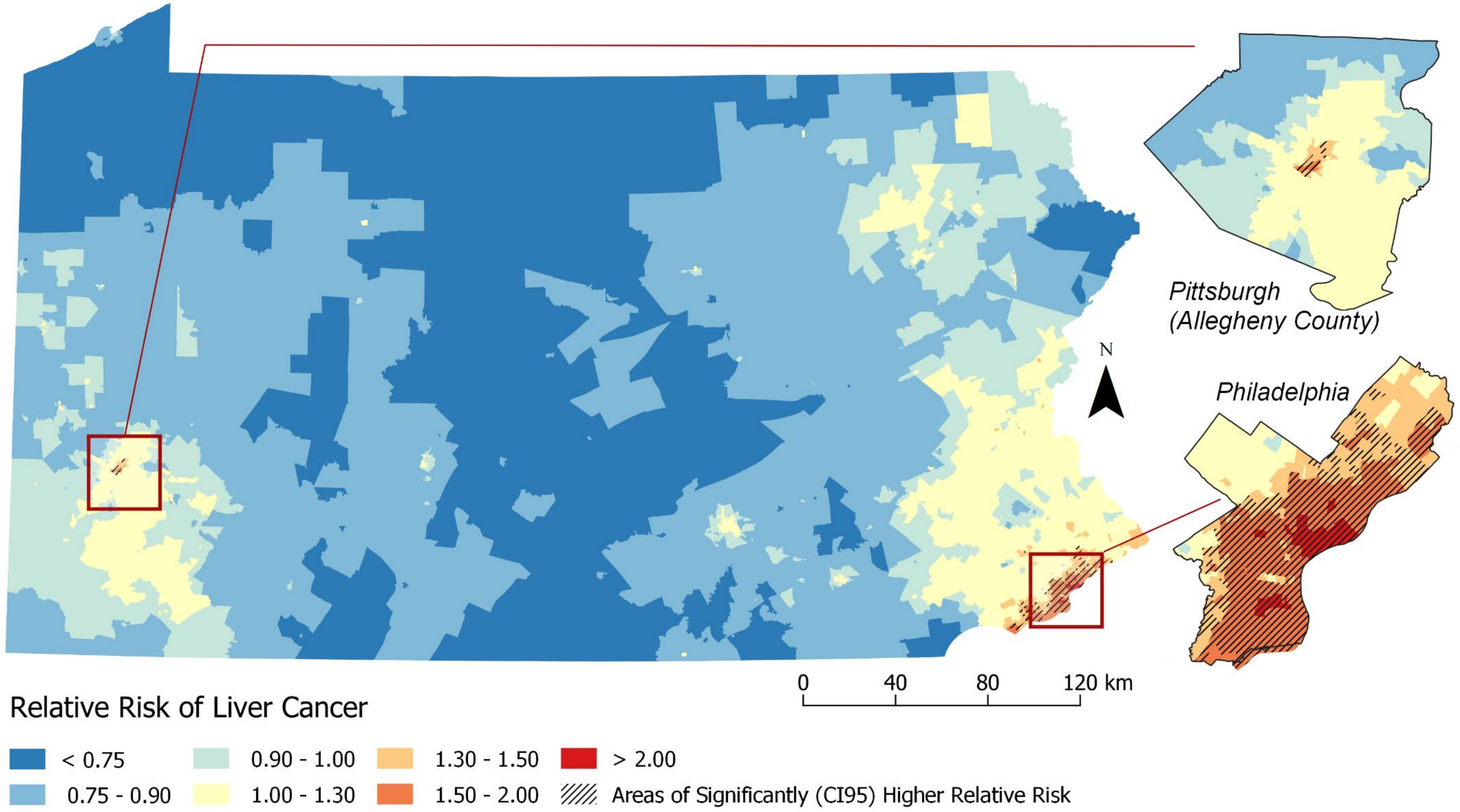

Figure S11. Relative Risk Estimates for Liver Cancer by Census Tract (Model 9. Adjusted for: individual-level factors + previous neighborhood variables (%Non-Hispanic Black (%NHB), Hispanic ICE, and Neighborhood Instability) + Messer); N = 283 Census Tracts within statistically significant high risk clusters for LC

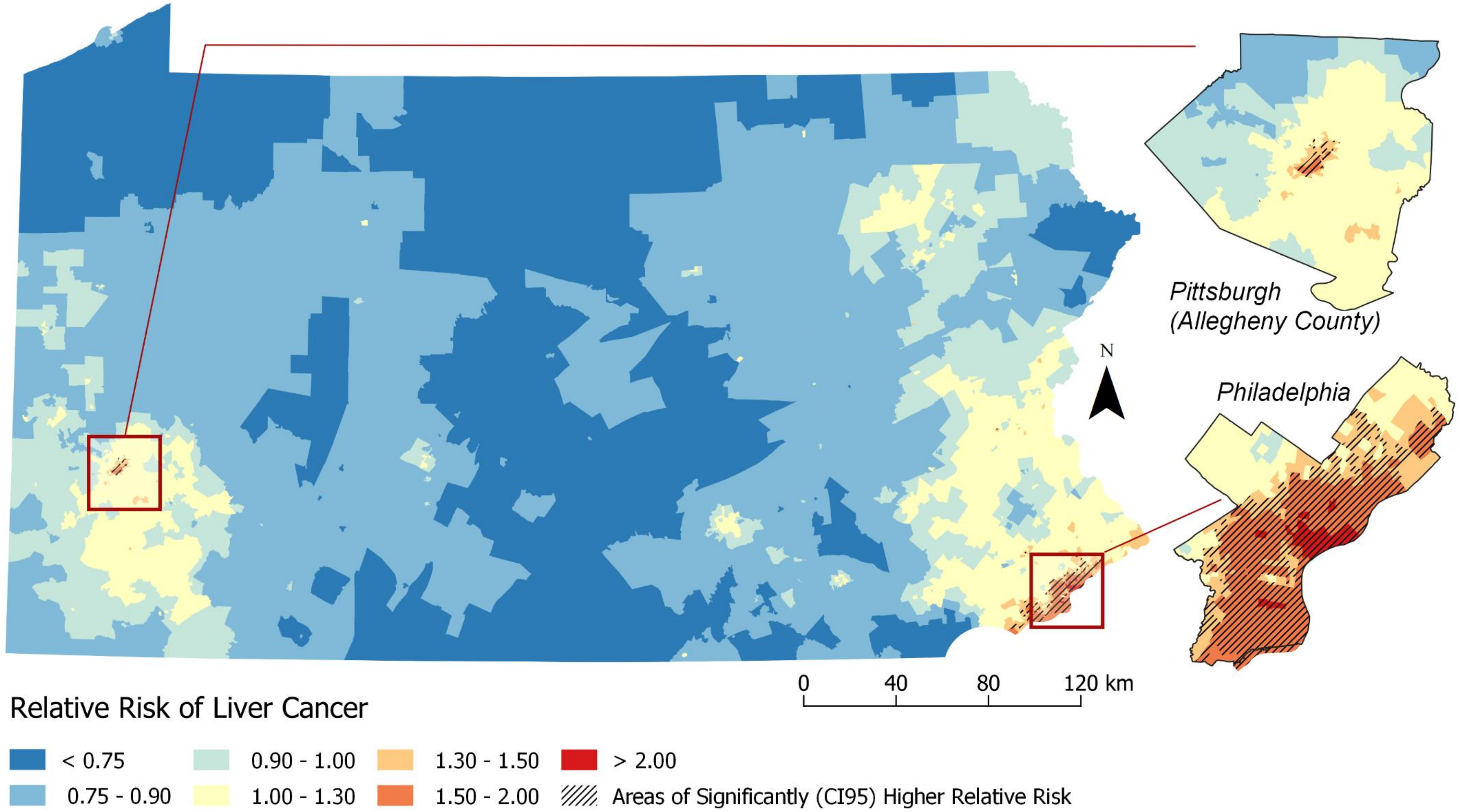

Figure S12. Relative Risk Estimates for Liver Cancer by Census Tract (Model 10. Adjusted for: individual-level factors + previous neighborhood variables (%Non-Hispanic Black (%NHB), Hispanic ICE, and Neighborhood Instability) +TDS/Yost); N = 322 Census Tracts within statistically significant high risk clusters for LC

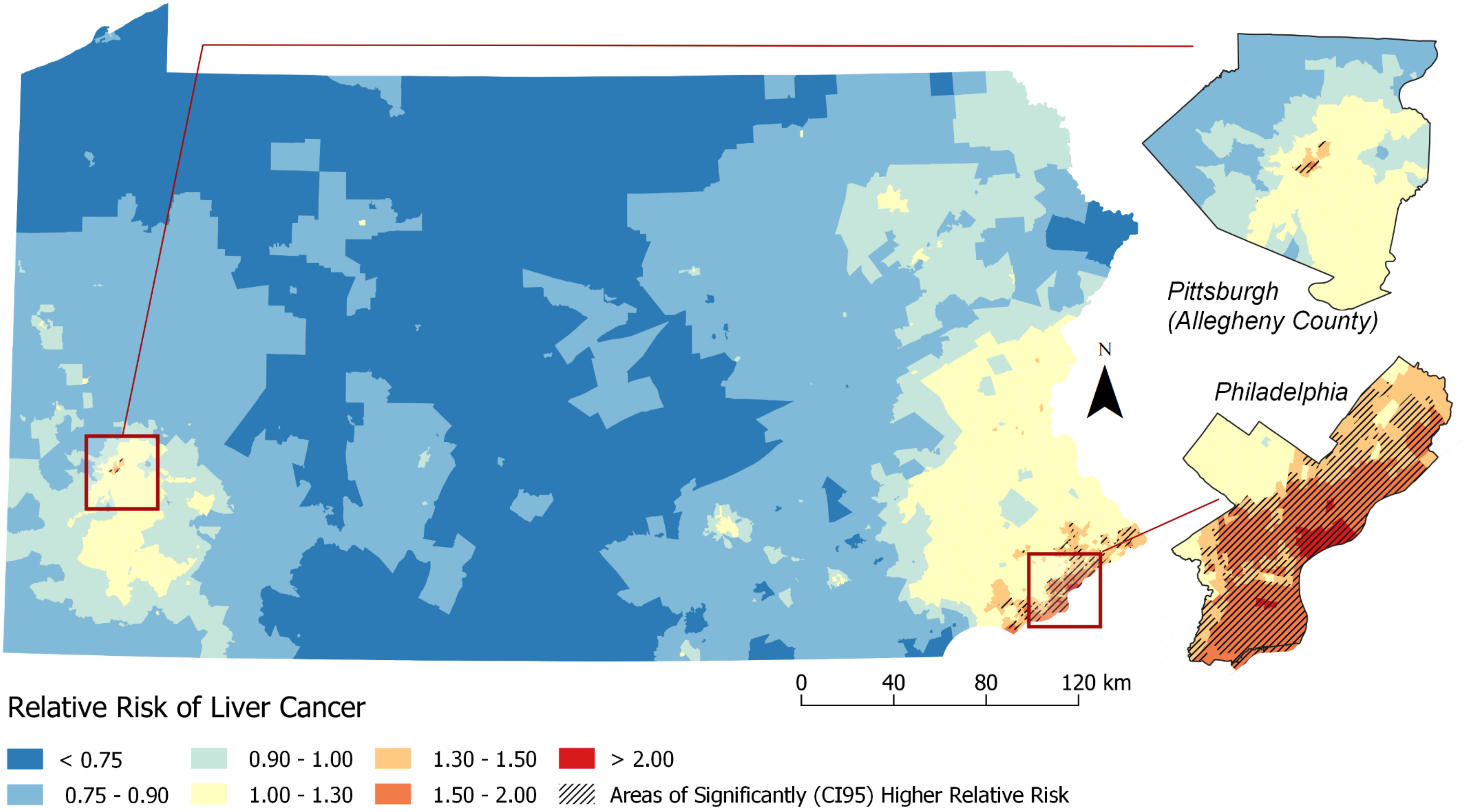

Figure S13. Relative Risk Estimates for Liver Cancer by Census Tract (Model 11. Adjusted for: individual-level factors + previous neighborhood variables (%Non-Hispanic Black (%NHB), Hispanic ICE, and Neighborhood Instability) + ICE-Income/Yost) N = 386 Census Tracts within statistically significant high risk clusters for LC

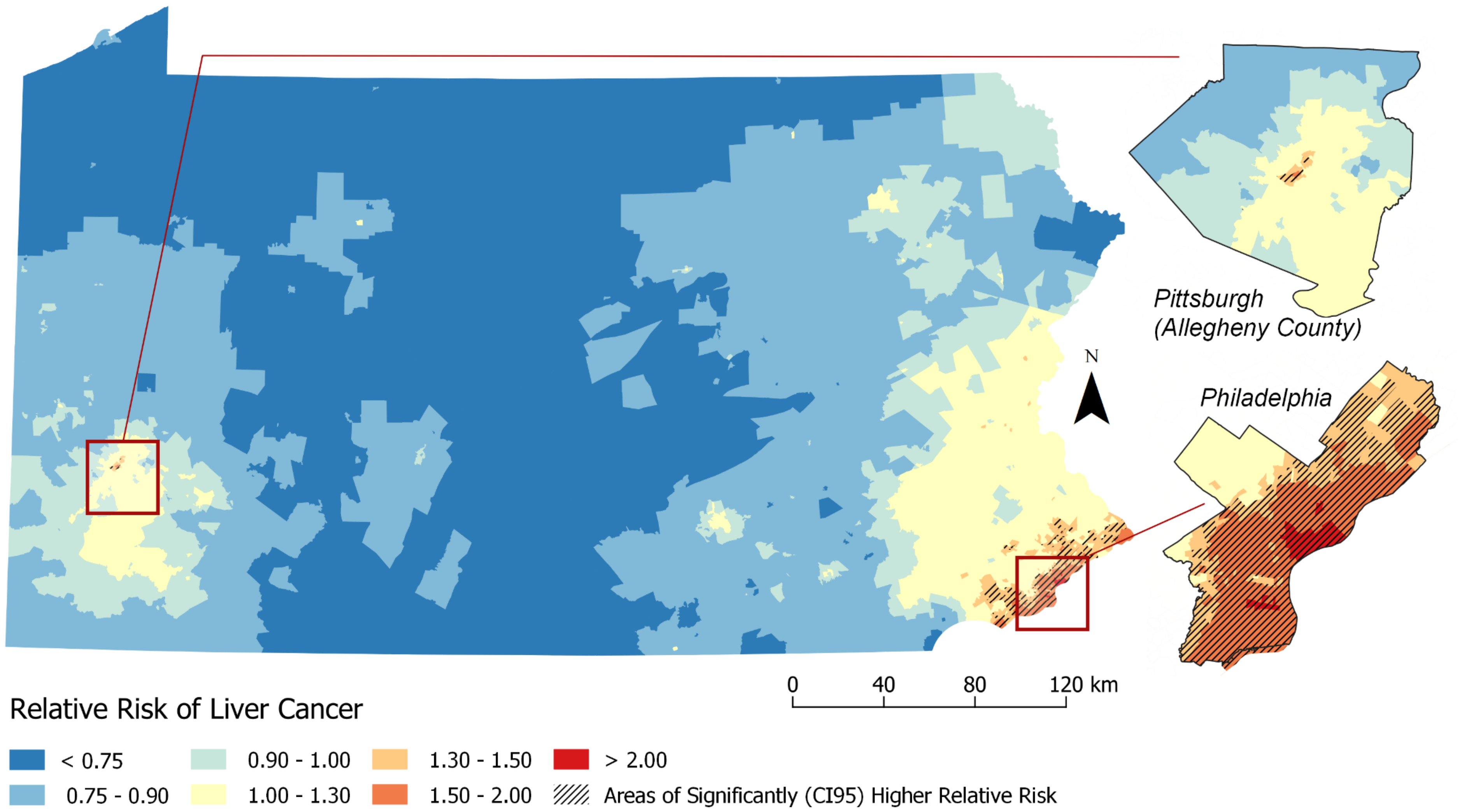

Figure S14. Relative Risk Estimates for Liver Cancer by Census Tract (Model 12. Adjusted for: individual-level factors + previous neighborhood variables (%Non-Hispanic Black (%NHB), Hispanic ICE, and Neighborhood Instability) +MESA/Yost); N = 377 Census Tracts within statistically significant high risk clusters for LC

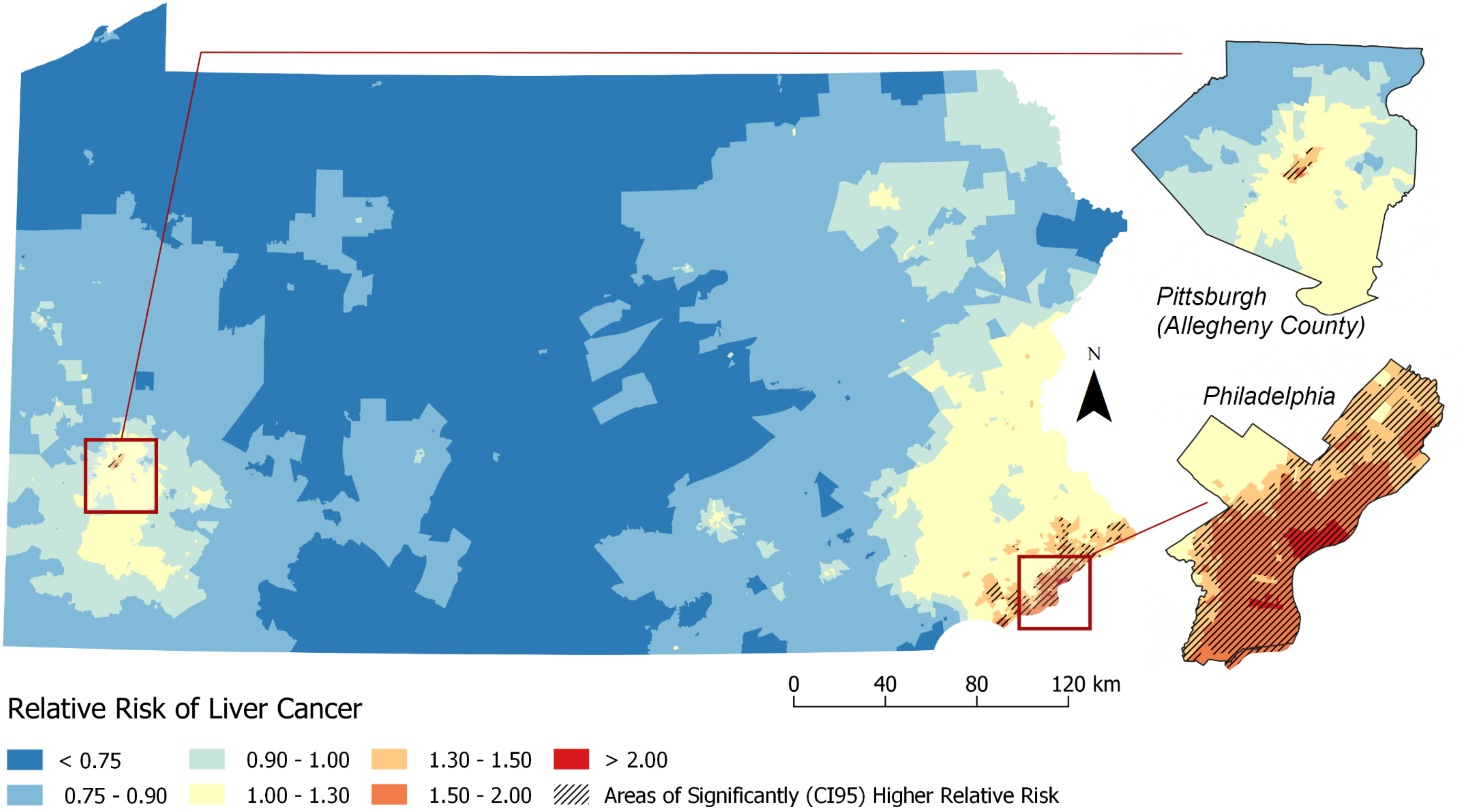

Figure S15. Relative Risk Estimates for Liver Cancer by Census Tract (Model 13. Adjusted for: individual-level factors + previous neighborhood variables (%Non-Hispanic Black (%NHB), Hispanic ICE, and Neighborhood Instability) +MESA/Messer; N = 296 Census Tracts within statistically significant high risk clusters for LC

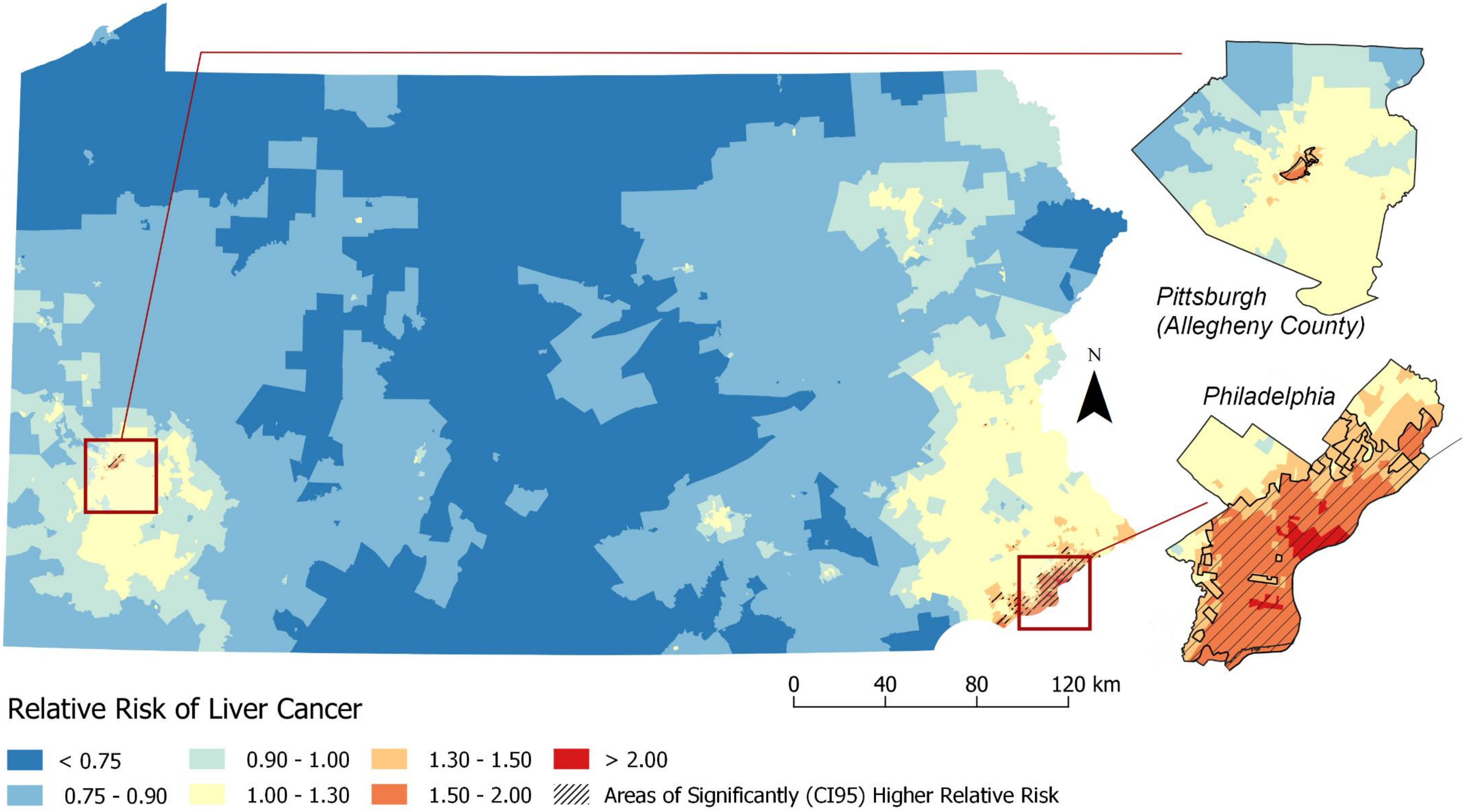

Table S1: Model Characteristics Comparing Model Fit (DIC), Relative Risk Range (RR), and Unexplained Geographic Disparity (GD) Percentage

	DIC	GD	RR Range	# CTs
**Model 1 (Individual-level)**	88,005	76.45%	0.37-4.03	370
**Model 2 (Individual-level + previous neighborhood variables)**	87,427	58.49%	0.46-3.44	195
**Model 3 (Poverty)**	87,608	47.56%	0.55-2.86	221
**Model 4 (ICE-Income)**	87,606	39.98%	0.58-2.53	319
**Model 5 (Townsend)**	87,513	48.19%	0.52-2.69	177
**Model 6 (Yost Index)**	87,558	40.71%	0.55-2.55	342
**Model 7 (SEP)**	87,393	54.91%	0.44-3.05	221
**Model 8 (MESA)**	87,591	42.25%	0.56-2.68	291
**Model 9 (Messer)**	87,535	43.02%	0.56-2.46	283
**Model 10 (Townsend/Yost Index)**	87,543	38.53%	0.58-2.44	322
**Model 11 (ICE-Income/Yost Index)**	87,617	37.84%	0.57-2.48	386
**Model 12 (MESA/Yost Index)**	87,606	38.63%	0.56-2.48	377
**Model 13 (MESA/Messer)**	87,525	40.34%	0.57-2.42	296

Abbreviations: DIC, deviance information criterion (model fit), lower values are better fit; GD, geographic disparity: square root of the spatial variance; disparity in relation to statewide relative risk average; lower number represent reduced disparities; RR Range, relative risk range previous neighborhoods variables; ICE, Index of Concentration at the Extremes; SES, Socio-economic status; SEP, Socio-economic position; MESA, Multi-Ethnic Study of Atherosclerosis; CT, census tracts; individual-level: age, sex, year of diagnosis, race; previous neighborhood variables: %Non-Hispanic Black (%NHB), Hispanic ICE, and Neighborhood Instability.
